# The efficacy and safety of immune checkpoint inhibitors for patients with EGFR‐mutated non‐small cell lung cancer who progressed on EGFR tyrosine‐kinase inhibitor therapy: A systematic review and network meta‐analysis

**DOI:** 10.1002/cam4.6453

**Published:** 2023-08-16

**Authors:** Zhen Wang, Fang Zhou, Shan Xu, Kang Wang, Huan Ding

**Affiliations:** ^1^ Department of Radiotherapy The Affiliated Yantai Yuhuangding Hospital of Qingdao University Yantai China; ^2^ Department of Oncology Zaozhuang Municipal Hospital Zao Zhuang China

**Keywords:** adverse events, efficacy, epidermal growth factor receptor, immune checkpoint inhibitors, network meta‐analysis, non‐small cell lung cancer

## Abstract

**Background:**

Non‐small cell lung cancer (NSCLC) patients harboring epidermal growth factor receptor (EGFR)‐mutated who progressed on EGFR tyrosine‐kinase inhibitor (EGFR‐TKI) therapy have limited therapeutic options. There is still no consensus on the role of immune checkpoint inhibitors (ICIs) in NSCLC with EGFR mutations.

**Methods:**

We did a network meta‐analysis (NMA) with a systematic literature search on PubMed, Embase, Web of Science, and The Cochrane Library. We included all phase II and III randomized controlled trials (RCTs), non‐randomized controlled trials (Non‐RCTs), and retrospective studies. Progression‐free survival (PFS) and overall survival (OS) were assessed through hazard ratios (HR). Objective response rate (ORR) and adverse events (AEs) were assessed through odds ratio (OR) and relative risk (RR), respectively. R software was used to compare the outcomes of different treatments by Bayesian NMA.

**Findings:**

We identified 1835 published results and 17 studies were included ultimately. A total of 2085 patients were included and accepted the following six treatments: ICIs plus chemotherapy (ICIs+Chemo), chemotherapy (Chemo), ICIs monotherapy (ICIs), ICIs plus chemotherapy and antiangiogenic therapy (ICIs+Chemo+Antiangio), antiangiogenic therapy plus chemotherapy (Antiangio+Chemo), ICIs plus antiangiogenic therapy (ICIs+Antiangio). ICIs+Chemo+Antiangio was associated with longer PFS and OS, as well as higher ORR (surface under the cumulative ranking curve [SUCRA], 96%, 90%, 91%). ICIs conferred the safety profile in terms of any‐grade AEs, grade greater than or equal to 3 AEs and any grade leading to treatment discontinuation occurred AEs (SUCRA, 99%, 68%, 94%).

**Interpretation:**

ICIs+Chemo+Antiangio brings the greatest survival benefit in NSCLC patients with EGFR mutations who progressed on EGFR‐TKI therapy, even for whom with baseline brain metastases. Compared with chemotherapy, ICIs has a low incidence of AEs and a benefit in OS.

## Graphical abstract

There is no consensus on the role of immune checkpoint inhibitors in NSCLC with EGFR mutations who progressed on EGFR tyrosine‐kinase inhibitor therapy. We did a network meta‐analysis with the result that ICIs plus Chemotherapy and Antiangiogenic therapy brings the greatest survival benefit, even for whom with baseline brain metastases. Compared with chemotherapy, ICIs had the highest safety.

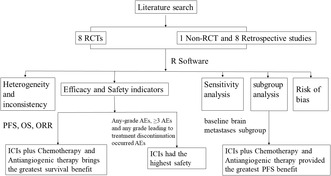

## 1 INTRODUCTION

Cancer has emerged as a major public health issue globally, lung cancer is the leading cause of cancer death in both sexes.^1^ By 2025, there are expected to be 2.52 million new cases of lung cancer worldwide,^2^ of which approximately 84% will be non‐small cell lung cancer (NSCLC).^3^ Most cases of NSCLC present with locally advanced or metastatic lesions at diagnosis^4^ with little opportunity for surgery, which result in a low overall 5‐year relative survival rate and poor prognosis.^5^, ^6^

In the age of precision medicine, a number of international studies have shown that targeted therapy can greatly improve the prognosis of NSCLC with corresponding driver genes.^7^, ^8^ For epidermal growth factor (EGFR) mutation‐positive advanced NSCLC, several classic clinical trials have shown that EGFR tyrosine‐kinase inhibitor (EGFR‐TKI) significantly improve survival,^9^, ^10^ establishing the status of EGFR‐TKI as the first‐line standard therapy. There are also many explorations on how to select targeted drugs and the deployment of targeted drugs. The longest progression‐free survival (PFS), 18.9 months, was obtained in the global multicenter Phase III FLAURA study of first‐line, third‐generation Osimertinib.^11^ Selecting third‐generation EGFR‐TKI after first‐line second‐generation EGFR‐TKI for Thr790Met mutation‐positive patients can achieve the longest PFS during targeted therapy, about 21–25 months.^12^, ^13^ For patients with first‐line first‐generation/second‐generation EGFR‐TKI progression without Thr790Met mutation, or first‐line third‐generation EGFR‐TKI failure, treatment with platinum‐containing double‐agent chemotherapy with or without bevacizumab is recommended, but with very limited survival benefit.^6^ Therefore, there is an urgent need to explore new treatment options for NSCLC patients with EGFR mutations who progressed after treatment with EGFR‐TKI.

In the era of immunotherapy, ICIs have shown promising results in NSCLC.^14^, ^15^ The phase III trial KEYNOTE‐189, demonstrated that pembrolizumab addition increased overall survival (OS) by 11.3 months compared with chemotherapy, establishing immunologic addition to platinum‐dual chemotherapy as first‐line therapy in metastatic NSCLC, but excluded patients with EGFR or ALK mutations.^16^, ^17^ The Phase III trial IMpower130 is of a similar design.^18^ Multiple clinical trials have shown that first‐line and second‐line ICIs does not improve survival in patients with EGFR mutations.^19^, ^20^ Even in patients whose PD‐L1 expressed high (>50%), the response rate of EGFR mutation was lower than that of the EFGR wild type.^21^ Can EGFR sensitive mutations be a negative factor in immunotherapy efficacy? ATLANTIC is the first study to study the efficacy of durvalumab in driver positive NSCLC, the results showed that patients with EGFR or ALK mutations had at least no lower OS than patients without EGFR or ALK mutations, at least when treated with durvalumab monotherapy.^22^ In terms of combination therapy, the results of a global multi‐center, open phase III clinical trial IMpower150 were announced in the subgroup of EGFR sensitizing mutations who had been previously treated with one or more TKIs, the addition of atezolizumab improved overall survival,^23^ it should be noted that this regimen also contains bevacizumab, an anti‐angiogenic drug. Another Phase III randomized controlled clinical trial, ORIENT‐31, published second interim results showing chemotherapy based on the addition of sintilimab (PD‐1 inhibitor) and IBI305 (bevacizumab biosimilar) was able to provide a significant PFS benefit to patients with EGFR‐TKI progression in non‐squamous NSCLC with EGFR‐mutation, and the benefit continued because the OS endpoint is not achieved in the experimental group.^24^ The publication of the results of these two large clinical trials has given us more expectations for the application of ICIs in EGFR‐mutated NSCLC who progressed on EGFR‐TKI. Meanwhile, CheckMate‐722, which has attracted worldwide attention, also announced results at the ESMO Asia Conference in December 2022 that the addition of Nivolumab resulted in only a small PFS benefit which did not reach statistical significance when compared with chemotherapy alone.^25^ The value of ICIs in EGFR‐mutated NSCLC with disease progression following EGFR‐TKI needs to be further explored.

ICIs remain controversial in NSCLC with EGFR mutations, particularly in patients with EGFR‐TKI progression, of which with limited treatment option. Can ICIs addition provide a new therapeutic direction? Therefore, we aimed to evaluate the efficacy and safety of ICIs addition in patients with EGFR‐mutated NSCLC who progressed on EGFR‐TKI therapy, and to conduct a systematic review and network meta‐analysis (NMA) to provide reference for clinical practice and future research.

## 2 METHODS

### 2.1 Protocol and registration

This NMA was conducted in accordance with the Preferred Reporting Items for Systematic Reviews and Meta‐Analyses 2020 statement for NMA (Supplement 1 in Appendix S1) and has been registered in the Prospective Register of Systematic Reviews (ID: CRD42023395779). Does not require institutional review board or ethics committee approval.

### 2.2 Search strategy

We searched PubMed, Embase, Web of Science, and The Cochrane Library for articles published before February 1, 2023 (Supplement 2 in Appendix S1). Congress abstracts were filtered in Embase and Web of Science based on the retrieval strategy. If there are multiple reports on the same study, the latest data was included. Ongoing or completed relevant trials were screened through the clinicaltrials.gov database simultaneously. The database retrieval was performed by ZW and FZ independently.

### 2.3 Inclusion and exclusion criteria

The inclusion criteria: (1) Locally advanced or metastatic NSCLC with sensitive EGFR mutations. (2) Progression occurred after EGFR‐TKI therapy. (3) Progression after receiving first/second‐generation EGFR‐TKI without EGFR Thr790Met mutation or after receiving third‐generation EGFR‐TKI as first/second‐line treatment was defined as disease progression. (4) Patients were treated with Programmed Cell Death‐Ligand 1 (PD‐L1), Programmed Cell Death Protein 1 (PD‐1), or cytotoxic T‐lymphocyte antigen‐4. (5) Randomized controlled trials (RCTs), non‐randomized controlled trials (Non‐RCTs), and retrospective studies. (6) Full‐text article or conference abstract. (7) English literature. (8) The study outcome including PFS, objective response rate (ORR), OS, adverse events (AEs) could be obtained directly or by calculation in this paper.

The exclusion criteria: (1) Inadequate date on endpoints. (2) Single arm or phase I studies. (3) Trial design schemes. (4) Case reports. (5) Systematic reviews.

### 2.4 Data extraction and management

Two reviewers (ZW and FZ) extracted the data using an electronic form independently. Any disagreements should be negotiated and agreed upon with HD. Detailed data included in the NMA were listed as follows:

- Study (Author, Year, Registered ID, Phase).
- Study type.
- Publication type.
- Race/Ethnicity.
- Mean age (years).
- Study period.
- Total patients (Male/Female).
- Number (No.) patients with EGFR‐mutated who resistance on EGFR‐TKI.
- Groups with EGFR‐mutated who resistance on EGFR‐TKI.
- No. patients with EGFR‐mutated who resistance on EGFR‐TKI in each group.
- Median follow‐up months.

Hazard ratios (HRs) and their standard error (SE) were used to evaluate the OS and PFS, which was extracted or calculated as follows:

1. If there are both unadjusted and adjusted statistics in the report, we extracted the adjusted data. For multi‐arm trials, we performed the analysis by calculating the SE of the control group according to the formula provided by Woods et al.^26^
2. If the HR was not reported directly but a survival curve with an at‐risk table was present in the article, the HR and its 95% confidence interval (CI) were calculated through the electronic computing table designed by Tierney et al.^27^

The ORR and AEs used dichotomous outcomes. Odds ratio (OR) and relative risk (RR) were used to evaluate ORR and AE, respectively.

### 2.5 Risk of bias

Revised Cochrane risk‐of‐bias tool (RoB 2) was used to evaluated the quality of RCTs.^28^ The assessment scale included the risk of bias due to randomization, deviations from intended intervention, missing data, outcome measurement, and selection of reported results. Each entry was judged as one of “Low,” “High,” or “Some concerns.” If all of the items have low risk scores, the literature was judged as “Low,” if any item was high risk, it was judged as “High,” otherwise it was judged as “Some concerns.”

Non‐RCTs and retrospective studies were evaluated by the ROBINS‐I tool.^29^ The assessment scale was as follows: bias due to confounding, selection of participants, classification of interventions, deviations from intended interventions, missing data, measurement of outcomes, selection of the reported result. Each entry was judged as one of “Low,” “Moderate,” “Serious,” and “Critical.” If all items were judged as low bias, the overall risk of the article was “Low.” If all items were judged as low or medium bias, the overall risk of the article was “Moderate.” At least one item was judged as serious and no critical, the article was judged as “Serious.” At least one item was judged as critical, and the article was judged as “Critical.”

### 2.6 Data synthesis and statistical analysis

Efficacy was evaluated by PFS (survival from study entry to disease relapse, or as defined by investigators), ORR (percentage of patients who reached an objective response, or as defined by investigators), OS (survival from study entry until death from all causes, or as reported by investigators), while safety indicators were determined according to Common Terminology Criteria for Adverse Events (CTCAE) criteria, focusing on AEs, including any‐grade, grade greater than or equal to 3, and any grade leading to treatment discontinuation occurred. Since we had no restrictions on the type of study, RCTs, Non‐RCTs, and retrospective studies were included. Each observation index was evaluated through RCTs data, which was verified by combined results including RCTs, Non‐RCTs, and retrospective studies. The final ranking of the treatment was evaluated using SUCRA.

We chose Bayesian NMA in R software (Appendix S2). Bayesian NMA provides a straightforward approach to probabilistic statements and predictions about treatment effects.^30^

Heterogeneity of treatment effects across studies was measured using the *I* ^2^ statistic. If *I* ^2^ ≥ 50%, the random effect model is used to evaluate the pool effect size; If *I* ^2^ < 50%, the fixed effect model is used.

Two sensitivity analyses and three subgroup analyses were selected to evaluated the stability and reliability of the results.

## 3 RESULTS

### 3.1 Systematic review and detailed data of the included studies

**FIGURE 1:**
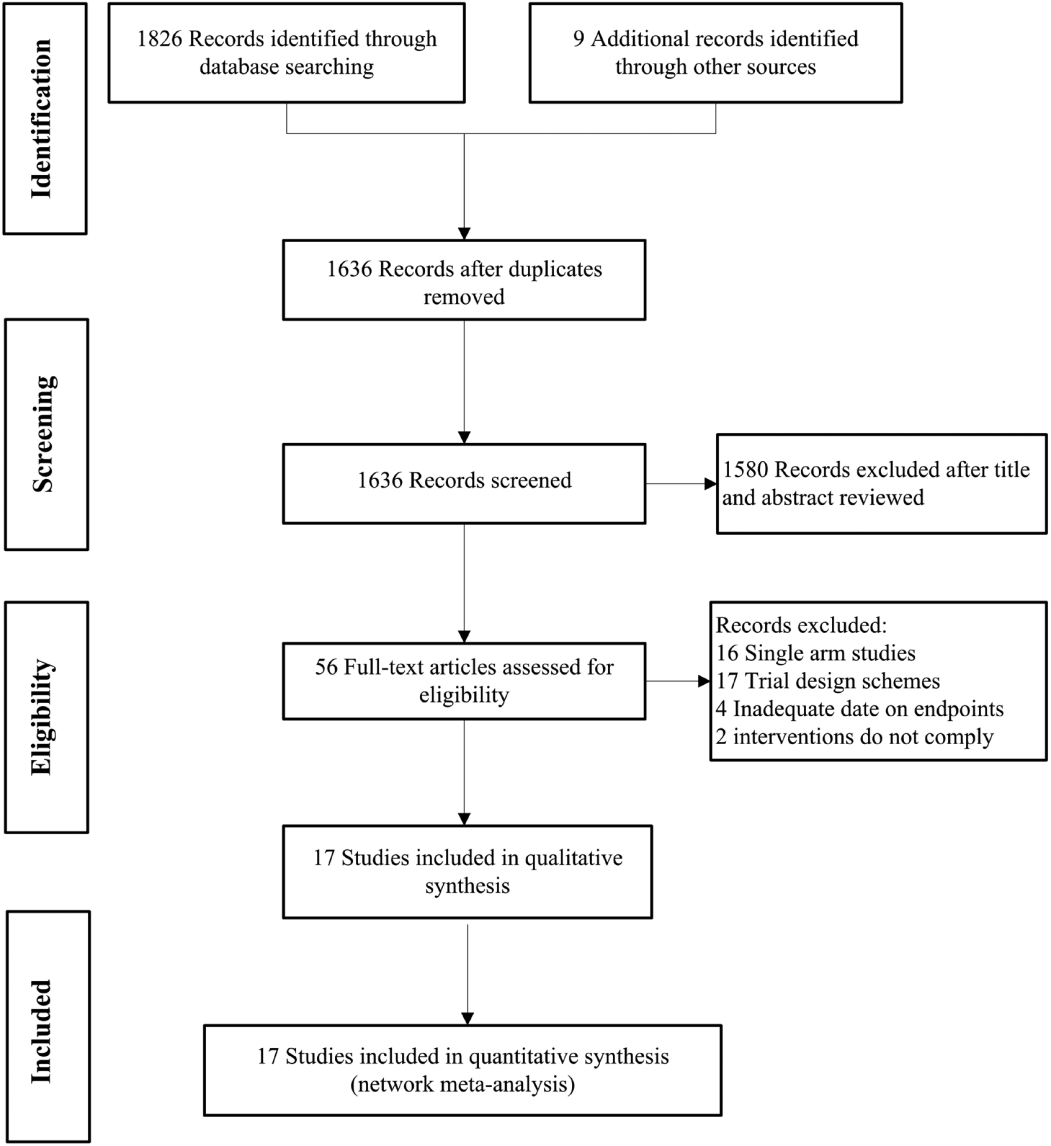
Preferred reporting items for systematic review and meta‐analysis.

During initial literature retrieval, 1826 records were screened from the database and nine additional articles were identified through other sources. About 56 studies were deemed eligible for full‐text review. Eventually, 17 studies were in accordance with our inclusion criteria (Figure 1), including eight RCTs,^23^, ^24^, ^25^, ^31^, ^32^, ^33^, ^34^, ^35^, ^36^, ^37^ eight retrospective studies,^38^, ^39^, ^40^, ^41^, ^42^, ^43^, ^44^, ^45^ and one Non‐RCT.^46^ Among them, the randomized controlled clinical trial IMpower150 data came from two literatures, the PFS data came from the literature with Martin Reck as the first author, and the OS data came from the literature with Naoyuki Nogami as the first author. The sample size ranged from 18 to 444 participants, and the total number of participants enrolled in the RCTs was 1132, the number of participants in the Non‐RCT and retrospective studies was 953. Finally, 2085 patients accepted the following six treatments: ICIs+Chemo, Chemo, ICIs, ICIs+Chemo+Antiangio, Antiangio+Chemo, ICIs+Antiangio. ICIs includes Pembrolizumab, Nivolumab, Atezolizumab, Durvalumab, Sintilimab, Camrelizumab, Toripalimab, Tislelizumab. Chemotherapy consists of drugs docetaxel, carboplatin, pemetrexed, cisplatin, paclitaxel, gemcitabine, platinum, navelbine, single, or combination chemotherapy. Antiangiogenic therapy included bevacizumab and its biosimilar (IBI305) (Table 1).

**TABLE 1:** Baseline characteristics of studies included in the NMA.

Study (Author, Year, Registered ID, Phase)	Study type	Publication type	Race/Ethnicity	Mean age (years)	Study period	Total patients (Male/Female)	Patients with EGFR‐mutated who resistance on EGFR‐TKI	Groups with EGFR‐mutated who resistance on EGFR‐TKI	No. Patients	Median follow‐up months
Herbst et al. 2015, NCT01905657, II/III	RCT	Full text	White/Asian/Black or African American/Other/Unknown	63.0	2013.08.28–2015.02.27	1034 (634/400)	86	Pembrolizuma	60	13.1
								Docetaxel	26	
Rittmeyer et al. 2016, NCT02008227, III	RCT	Full text	White/Asian/Black/Other/Unknown	64·0	2014.03.11–2014.11.28	850 (520/330)	85	Atezolizumab	42	21.0
								Docetaxel	43	
Fehrenbacher et al. 2016, NCT01903993, II	RCT	Full text	NG	62.0	2013.08.05–2014.03.31	287 (169/118)	18	Atezolizumab	10	14.8
				62.0				Docetaxel	8	15.7
Arrieta et al. 2020, NCT02574598, II	RCT	Full text	NG	50.1	2016.12–2019.05	78 (31/46)	25	Pembrolizumab +Docetaxel	12	8.9
				62.1				Docetaxel	13	7.9
Hayashi et al. 2022, UMIN000001919/jRCTs051180133, II	RCT	Full text	Asian	70.5	2016.04–2019.06	102 (41/59)	102	Nivolumab	52	25.5
				67.0				Carboplatin–pemetrexed	50	23.4
Lu et al. 2022, NCT03802240, III	RCT	Full text	Asian	59.0	2019.07.11–2021.07.31	444 (183/261)	444	Sintilimab+IBI305+pemetrexed+cisplatin	148	13.1
				57.0				Sintilimab+pemetrexed+cisplatin	145	
				56.0				Pemetrexed+cisplatin	151	
Nogami et al. 2019, 2022, NCT02366143, III	RCT	Full text	White/Asian/Black/American Indian or Alaska/Native/Multiple/Unknown	64.0	2015.03.31–2016.12.30	1202 (720/482)	78	Atezolizumab+bevacizumab+carboplatin/paclitaxel	22	39.3
				63.0				Atezolizumab+carboplatin/paclitaxel	28	
				61.5				Bevacizumab+carboplatin/paclitaxel	28	
Mok et al. 2022, NCT02864251, III	Conference abstract	Full text	NG	NG	NA	294 (NG)	294	Nivolumab+ Pemetrexed+Cisplatin/Carboplatin	144	18.2
								Pemetrexed+Cisplatin/Carboplatin	150	
White et al. 2021	Retrospective study	Full text	Asian/White/Other	62.9	2020.07.05–2021.04.09,^a^ 2020.12.04–2021.04.09^b^	104 (37/67)	104	Chemo‐IO	12	NG
				56.5				Chemotherapy	57	
				60.9				Chemo‐Bev	35	
Hu et al. 2021	Retrospective study	Full text	Asian	NA	2018.03–2019.12	56 (31/25)	56	PD‐1 inhibitors combined with chemotherapy	21	NG
								PD‐1 inhibitors combined with bevacizumab	8	
								PD‐1 inhibitors combined with chemotherapy and bevacizumab	20	
								PD‐1 inhibitors	7	
Shen et al. 2021	Retrospective study	Full text	Asian	66.5	2014.01–2019.12	30 (13/17)	30	ICI+Chemo	8	16.76
								ICI	22	
Lu et al. 2022	Retrospective study	Full text	Asian	58.30	2018.09.01–2021.03.01	64 (27/37)	64	Pembrolizumab	32	NG
								Pemetrexed plus platinum	32	
Zhou et al. 2022	Retrospective study	Full text	Asian	NA	2019.03–2021.09	93 (27/26)	93	ICI plus chemotherapy plus antiangiogenic	18	6.9
								ICI plus chemotherapy	19	
								ICI plus antiangiogenic	12	
								ICI therapy	4	
								Chemotherapy	40	
Cheng et al. 2022	Retrospective study	Full text	Asian	57.0	2018.09–2020.07	132 (60/72)	132	ICI+Chemotherapy	61	21.7
								ICI	11	
								Chemotherapy	60	
Hong et al. 2022	Retrospective study	Full text	Asian/Black/Hispanic/Latino/White	63.3	2014.03–2021.03	178 (75/103)	178	ChemoBevIO	11	42.0
								ChemoIO	30	
								IO‐mono	22	
								ChemoBev	31	
								Chemo	84	
Bylicki et al. 2023	Non‐RCT	Full text	NG	60.4	2019.09–2021.10	150 (NG)	132	Atezolizumab+bev acizumab+platinum+pemetrexed	62	14.8
				66.1				Platinum+pemetrexed	70	13.1
Chen et al. 2023	Retrospective study	Full text	Asian	65.5	2015.01.01–2021.12.01	164 (65/99)	164	Pembrolizumab+chemotherapy	82	12.5
				59.0				Chemotherapy	82	13.1

Abbreviations: ChemoBev, chemotherapy plus bevacizumab without immunotherapy; ChemoBevIO, chemotherapy plus bevacizumab and Immunotherapy; ChemoIO, chemotherapy plus immunotherapy; ICI+C, immunotherapy in combination with chemotherapy.

^a^ Stanford Cancer Institute.

^b^ Massachusetts General Hospital.

Since chemotherapy is the most common comparator in RCTs and the standard treatment for NSCLC with EGFR mutations progression after prior EGFR‐TKI therapy, all treatments were compared with chemotherapy. All treatments were also compared with single agent ICIs to know if combination therapy with ICIs was superior to monotherapy.

### 3.2 Risk of bias

The results were provided in Supplement 3 in Appendix S1. In the RCTs study, 4 and 3 were assessed as “Low” and “Some concerns,” respectively. Due to the limited information contained in the conference abstract, CheckMate‐722 was assessed to be “High.” In the Non‐RCT and retrospective studies, the study by Lu et al. was judged to be “Critical,” the studies by Bylicki et al. and White et al. were judged to be “Low,” the others were assessed as “Moderate” or “Serious.”

### 3.3 Heterogeneity and inconsistency assessment

We performed heterogeneity analysis of PFS, ORR, OS, and AEs, including any‐grade, grade greater than or equal to 3, and any grade leading to treatment discontinuation occurred. Most of the comparisons of these different observed indicators showed low heterogeneity. High heterogeneity (*I* ^2^ ≥ 50%) was detected in the comparison of following outcomes: ICIs versus Chemo in PFS (92.4%), Chemo versus ICIs+Chemo and ICIs+ Chemo+Antiangio versus ICIs+Chemo in AEs of grade greater than or equal to 3 (79.2%, 88.4%, respectively).

We used deviation information criteria (DIC) to measure the complexity of the model's fitting adjustment model to fit consistent and inconsistent models. We found that the consistency model of the NMA was similar to or better fitted to the inconsistency model (Supplement 4 in Appendix S1), representing the global consistency.

### 3.4 Comparison of efficacy indicators PFS, OS, and ORR

#### 3.4.1 PFS

**FIGURE 2:**
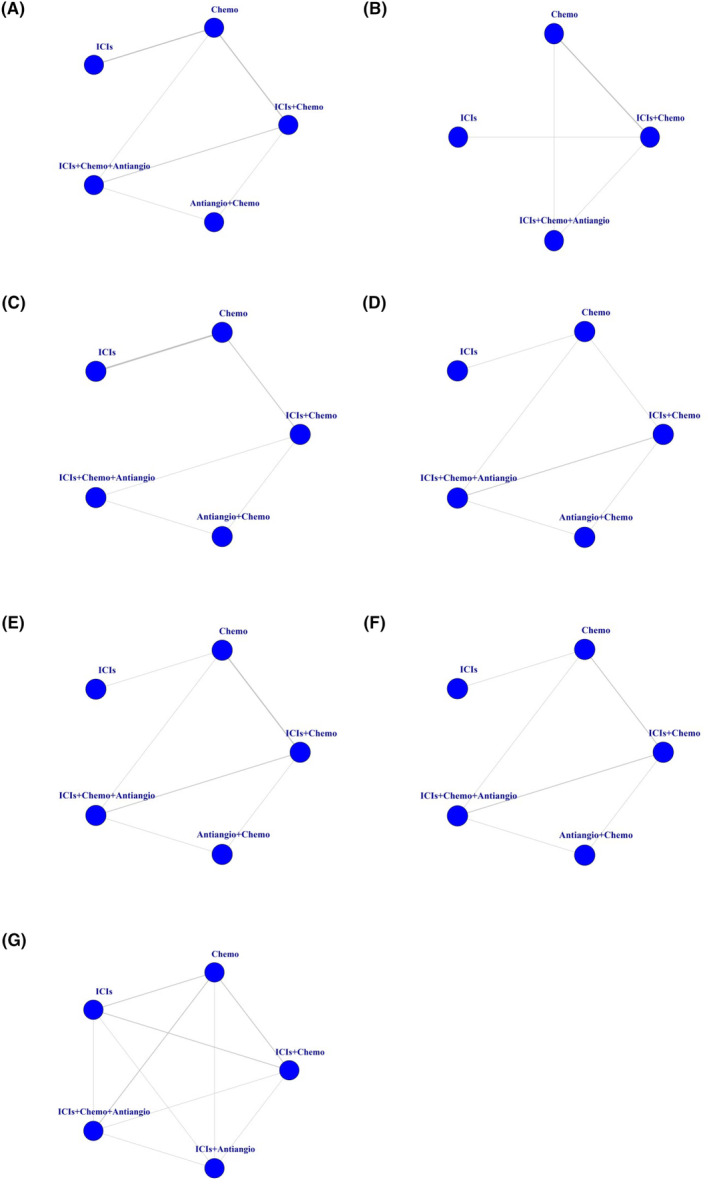
Comparative network plots for efficacy and toxicity of ICIs for patients with EGFR‐mutated NSCLC who progressed on EGFR‐TKI therapy. Comparisons were generated by using the Bayesian framework on. (A) PFS. (B) OS. (C) ORR. (D) Safety assessed according to AEs of any‐grade. (E) Safety assessed according to AEs of grade greater than or equal to 3. (F) Safety assessed according to AEs of any grade leading to treatment discontinuation occurred. (G) PFS for patients with baseline brain metastases. Each circle represents a treatment. AEs, adverse events; Antiangio+Chemo, antiangiogenic therapy plus chemotherapy; Chemo, chemotherapy; EGFR, epidermal growth factor; ICIs, immune checkpoint inhibitors; ICIs+Chemo, ICIs plus chemotherapy; ICIs+Chemo+Antiangio, ICIs plus chemotherapy and antiangiogenic therapy; ICIs+Antiangio, ICIs plus antiangiogenic therapy; NSCLC, non‐small cell lung cancer; OS, overall survival; ORR, objective response rate; PFS, progression‐free survival.

**FIGURE 3:**
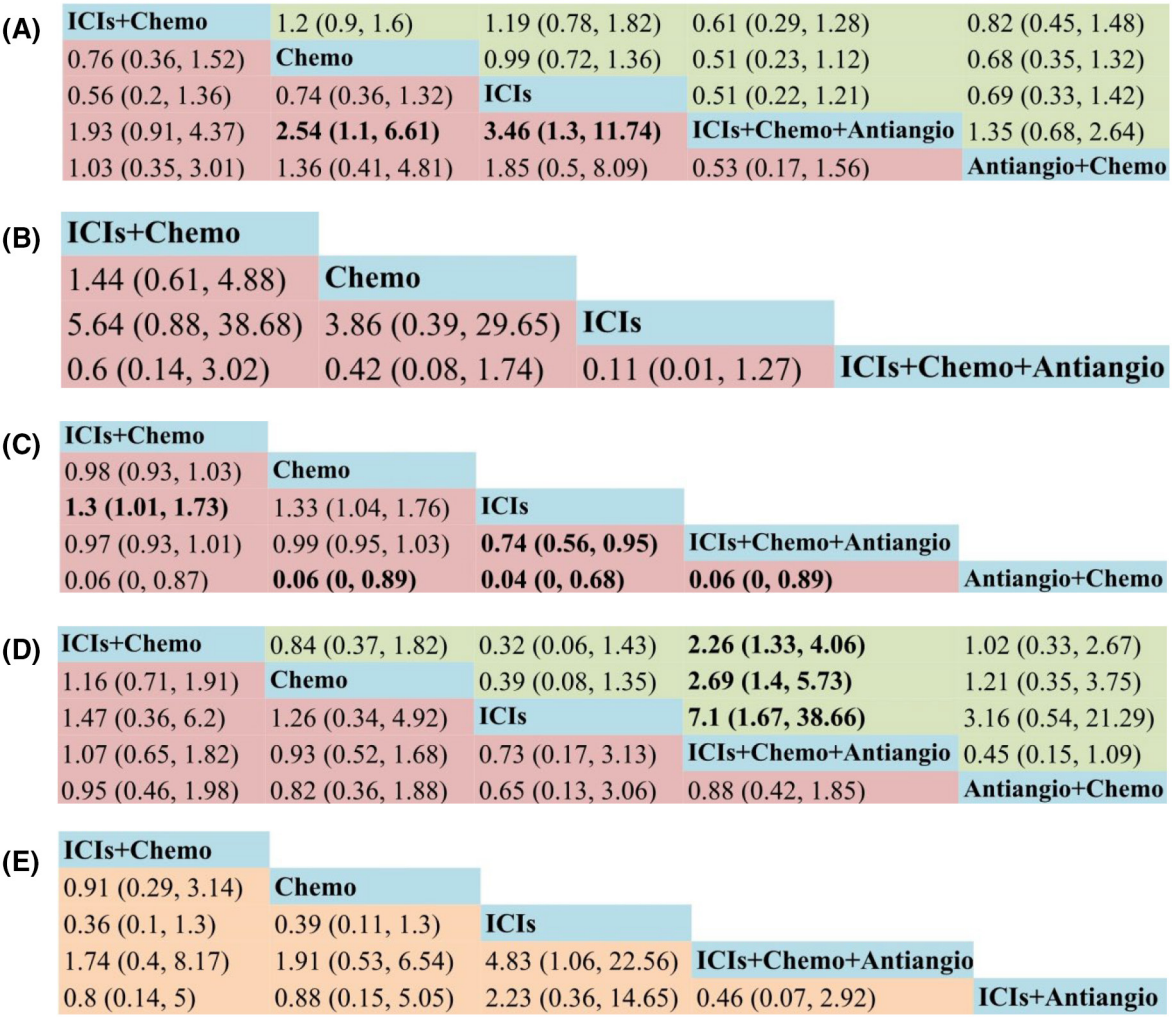
Pooled estimates of the network meta‐analysis. (A) Pooled HRs (95% CI) for OS in the upper triangle and PFS in the lower triangle. (B) Pooled ORs (95% CI) for ORR. (C) Pooled RRs (95% CI) for Safety assessed according to any grade AEs. (D) Pooled RRs (95% CI) for Safety assessed according to grade 3 or higher AEs in the upper triangle and any grade leading to treatment discontinuation occurred AEs in the lower triangle. (E) Pooled HRs (95% CI) for PFS of patients with baseline brain metastases subgroups. AEs, adverse events; Antiangio+Chemo, antiangiogenic therapy plus chemotherapy; CI, credible interval; Chemo, chemotherapy; HR, hazard ratios; ICIs, immune checkpoint inhibitors; ICIs+Chemo, ICIs plus chemotherapy; ICIs+Chemo+Antiangio, ICIs plus chemotherapy and antiangiogenic therapy; ICIs+Antiangio, ICIs plus antiangiogenic therapy; OS, overall survival; ORR, objective response rate; PFS, progression‐free survival; OR, odds ratio; RR, relative risk.

**FIGURE 4:**
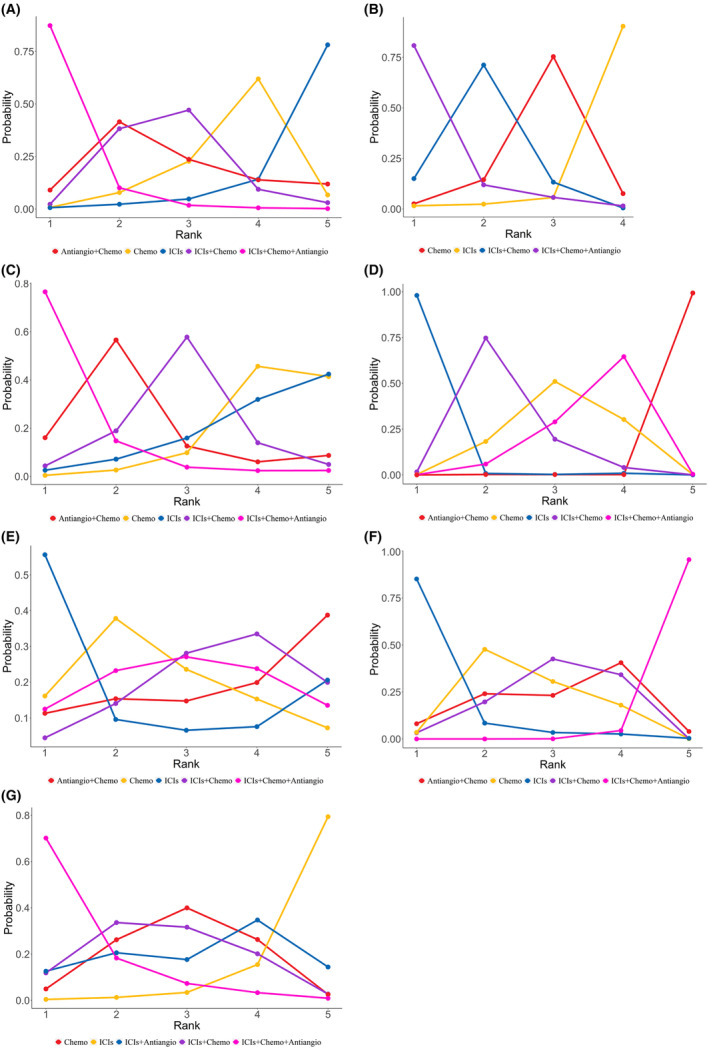
Bayesian ranking profiles of efficacy and safety of ICIs for patients with EGFR‐mutated NSCLC who progressed on EGFR‐TKI therapy. Profiles indicate the probability of each treatment being ranked from first to last on PFS, OS, ORR, and Safety assessed according to AEs. The horizontal coordinate represents the “Rank,” and the vertical coordinate represents the “Probability.” Different colored lines represent different interventions. The position on the vertical coordinate corresponding to each circle indicates the ranking probability of each intervention. (A) PFS. (B) ORR. (C) OS. (D) Safety assessed according to AEs of any‐grade. (E) Safety assessed according to AEs of grade greater than or equal to 3. (F) Safety assessed according to AEs of any grade leading to treatment discontinuation occurred. (G) Brain metastases at baseline. AEs, adverse events; Antiangio+Chemo, antiangiogenic therapy plus chemotherapy; Chemo, chemotherapy; EGFR, epidermal growth factor; ICIs, immune checkpoint inhibitors; ICIs+Chemo, ICIs plus chemotherapy; ICIs+Chemo+Antiangio, ICIs plus chemotherapy and antiangiogenic therapy; ICIs+Antiangio, ICIs plus antiangiogenic therapy; NSCLC, non‐small cell lung cancer; OS, overall survival; ORR, objective response rate; PFS, progression‐free survival.

A total of 15 studies were included, including seven RCTs, one Non‐RCT, and seven retrospective studies (Supplement 5 in Appendix S1). NMA included six treatments and seven studies (Figure 2A). The relative effects of RCTs were shown in Figure 3A. Compared with chemo, ICIs+Chemo+Antiangio group could significantly improve PFS (HR = 0.39, 95% CI 0.15–0.91) in RCTs, which was consistent with the combined results of PFS (HR = 0.45, 95% CI 0.28–0.73) (Supplement 6 in Appendix S1). There was no ICIs+Antiangio group in the RCTs, so the efficacy of ICIs+Antiangio versus chemotherapy group was derived from the combined results, which showed that ICIs+Antiangio could improve PFS, but not statistically significant. Compared with ICIs, the combination therapy of immunotherapy can improve PFS, but only achieved statistical significance at ICIs+Chemo+Antiangio (HR = 0.34, 95% CI 0.19–0.58), which was verified in the combined results. The PFS effect of the remaining pairwise regimens did not reach statistical difference. Ranking analysis based on SUCRA scores indicated that ICIs+Chemo+Antiangio (SUCRA, 96%) has the greatest likelihood of being the best choice in terms of PFS benefits, followed by ICIs+Chemo (SUCRA, 57%) (Figure 4A) (Supplement 7 in Appendix S1).

#### 3.4.2 ORR

ORR have been reported from four RCTs and four retrospective studies (Supplement 8 in Appendix S1). NMA included four treatments and four studies for ORR (Figure 2B). The relative effects of RCTs were shown in Figure 3B. In comparison with Chemo, both ICIs+Chemo and ICIs+Chemo+Antiangio could improve ORR, with no statistical difference in RCTs, while ICIs failed to improve the ORR. According to the ranking analysis of SUCRA score, ICI+Chemo+Antiangio (SUCRA, 91%) may be the best choice for ORR benefit (Figure 4B) (Supplement 7 in Appendix S1).

#### 3.4.3 OS

A total of 13 studies were included, including seven RCTs, one Non‐RCT, and five retrospective studies (Supplement 9 in Appendix S1). NMA included five treatments and seven studies for OS (Figure 2C). The relative effects of RCTs were shown in Figure 3A. In comparison with Chemo, OS was improved in either treatment group, but unfortunately no statistical difference was achieved in RCTs. Ranking analysis based on SUCRA score showed that ICI+Chemo+Antiangio (SUCRA, 90%) may be the best choice for OS benefit (Figure 4C) (Supplement 7 in Appendix S1). The results of the above RCTs on OS are consistent with the combined results (Supplement 6 in Appendix S1).

### 3.5 Safety and toxicity

NMA included three RCTs and two retrospective studies for any‐grade AEs, five RCTs and two retrospective studies for grade greater than or equal to 3 AEs, four RCTs and one retrospective study for any grade leading to treatment discontinuation occurred AEs (Supplement 10 in Appendix S1).

In terms of any grade AEs (Figure 2D), compared with Chemo, ICIs associated with minimal side effects and highest safety (RR = 0.75, 95% CI 0.58–94), and adding ICIs to chemotherapy (with or without antiangiogenic therapy) did not increase toxicity. When compared with immunotherapy alone, both the combination therapy groups and the chemotherapy group had increased toxicity (ICIs+Chemo vs. ICIs, RR = 1.3, 95% CI 1.01–1.73; ICIs+Chemo+Antiangio vs. ICIs, RR = 1.34, 95% CI 1.05–1.78; Chemo vs. ICIs, RR = 1.33, 95% CI 1.04–1.76) (Figure 3C).

In terms of grade 3 or higher AEs (Figure 2E), the results were consistent with any grade AEs, but no statistical difference was achieved (Figure 3D).

In terms of any grade leading to treatment discontinuation occurred AEs (Figure 2F), compared with Chemo, ICIs remained minimally toxic, Nevertheless ICIs+Chemo+Antiangio significantly increased AEs of any grade leading to treatment discontinuation occurred (RR = 2.69, 95% CI 1.4–5.73) (Figure 3D).

According to the SUCRA score, ICIs had the highest safety regardless of any grade AEs, grade greater than or equal to 3 AEs and any grade leading to treatment discontinuation occurred AEs (Figure 4D–F) (Supplement 7 in Appendix S1).

### 3.6 Baseline status subgroup analysis of PFS

#### 3.6.1 Baseline BMS subgroup analysis

In terms of PFS (Figure 2G), two RCTs and three retrospective studies were incorporated into analyzed (Supplement 11 in Appendix S1). Only two RCTs compared the efficacy of ICIs and ICIs+Chemo+Antiangio with chemotherapy, respectively, so we chose to combined results in real clinical settings (Figure 3E). Compared with Chemo, ICIs+Chemo and ICIs+Chemo+Antiangio could improve PFS, with no statistical difference. ICIs failed to improve the PFS. Compared with ICIs, both ICIs+Chemo and ICIs+Chemo+Antiangio showed PFS benefits in the BMS subgroup, and the difference was statistically significant in ICIs+Chemo+Antiangio (HR = 0.21, 95% CI 0.04–0.95). Based on the SUCRA score results (88%), the ICIs+Chemo+Antiangio group still provided the greatest PFS benefit (Figure 4G) (Supplement 7 in Appendix S1).

#### 3.6.2 Baseline immune status subgroup analysis

NMA included two RCTs and three treatments (Supplement 12 in Appendix S1). ICIs+Chemo improved PFS when compared with Chemo, regardless of PD‐L1 <1%, PD‐L1 1–49%, or PD‐L1 ≥50% subsets, while ICIs had the opposite effect. Compared with ICIs, both ICIs+Chemo and Chemo improved PFS with no statistical difference. The efficacy of ICIs+Chemo, Chemo, and ICIs was consistent with that of the total population (Supplement 13 in Appendix S1).

### 3.7 Population characteristics subgroup analysis

We analyzed the effect of gender and tobacco use history on PFS separately. There were two RCTs and one retrospective study in this NMA (Supplement 12 in Appendix S1). In all subset analyses, ICIs+Chemo and ICIs+Chemo+Antiangio showed PFS benefits compared with Chemo and ICIs, respectively, but the differences did not reach statistical significance (Supplement 14 in Appendix S1). Based on SUCRA scores, the ranking probability of PFS was consistent with that of the general population (Supplement 15 in Appendix S1).

### 3.8 Sensitivity analysis

To evaluate the robustness of the statistical inference, the following two sensitivity analyses were performed as followed: (i) the studies by Shun Lu and Naoyuki Nogami were removed because of three‐arm study, result was consistent with the original one, and ICIs+Chemo+Antiangio was still probably the best choice in the aspect of PFS benefits. (ii) the results of RCTs and combined results were analyzed, respectively, which showed that there was consistency among the observed indicators.

## 4 DISCUSSION

### 4.1 Principal findings

We included eight RCTs, one Non‐RCT, and eight retrospective studies involving 2085 NSCLC patients with EGFR mutations who progressed prior treatment with EGFR‐TKI and received ICIs alone or in combination with follow‐up therapy. Key discoveries are listed as follows: (1) ICIs+Chemo+Antiangio could be used as the first choice of treatment because it provided more survival benefit than immune monotherapy and chemotherapy regarding of PFS, OS, ORR, meanwhile the any grade AEs and grade 3 or higher AEs were tolerable. But any grade leading to treatment discontinuation occurred AEs should be vigilant. (2) The PFS benefit of ICIs+Chemo+Antiangio versus chemotherapy was consistent with subgroup of patients with BMS. (3) Neither ORR nor OS was statistically different among the currently included treatment regimens.

For patients who progress again after resistance to targeted therapy, the therapeutic regimens are very finite. The issue of progressing on EGFR‐TKI therapy for patients with EGFR mutations has also received increasing global attention. ICIs have shown great efficacy in NSCLC. Unfortunately, in the large clinical trials of immunotherapy in NSCLC, the positive driver genes are all excluded from the inclusion criteria. But what about the efficacy of ICIs after advances in targeted therapies? Several RCTs have been conducted in recent years to explore the best treatment options for such patients. However, due to the differences in study results, meta‐analyses are needed to explore the best treatment options, so we designed this NMA. There was a meta‐analysis before,^47^ but since then, large clinical trial data have been published and updated, and we have increased the number of RCTs through rigorous design and comprehensive literature search. At the same time, we included data from retrospective studies, which can better reflect the real clinical treatment environment.

Several studies have shown that EGFR‐TKI can cause some changes in the tumor immune microenvironment (TME) during the treatment of NSCLC. It can improve the immune response by increasing the infiltration of CD8+ T cells, inhibiting the infiltration of regulatory T cells,^48^ and up‐regulating the adaptive response triggered by type I interferon.^49^, ^50^ Changes in TME can also be detected after acquiring EGFR‐TKI resistance in NSCLC. Peng et al. found that c‐MET amplification and hepatocyte growth factor (HGF) can activate PI3K/Akt, MAPK, and other signaling pathways, and HGF can also regulate the proliferation of T lymphocytes and the secretion of cytotoxicity, which induce the expression of PD‐L1 in EGFR‐mutated NSCLC cells, thus promoting lung cancer immune escape.^51^ This result has also been verified in tissue specimens. Isomoto et al. found that the proportion of patients with high PD‐L1 expression (≥50%) after progression during EGFR‐TKI treatment increased from 14% to 28%, and TMB also showed an increasing trend (3.3 → 4.1108 mutations/Mbp, *p* = 0.0508) through rebiopsy of patients, so that it enters into the immune‐supportive TME, which may provide basis for the subsequent ICIs treatment optimization.^52^ Maynard et al. used single‐cell RNA sequencing to identify decreased T‐cell infiltration (46% vs. 31%) and increased macrophage infiltration (21% vs. 37%), promoting immune tolerance and revealing a transient immunosuppressive environment during TKI therapy, so single‐agent immunotherapy may not be able to overcome this complex immune escape.^53^ During tumor development, vascular abnormalities lead to hypoxia and acidosis in TME, which promote immune suppression by several mechanisms such as increasing the accumulation, activation, and expansion of immune suppressive regulatory T (Treg) cells, inhibiting of dendritic cell maturation, which results in impaired antigen presentation and activation of tumor‐specific cytotoxic T lymphocytes.^54^ Currently, many studies have shown that anti‐angiogenic agents could normalize tumor blood vessels, reverse VEGF‐mediated immunosuppression, and promote immune cell differentiation, thus enhancing the efficacy of immunotherapy, which has been clinically confirmed.^55^, ^56^ These results may provide a theoretical basis for the use of ICIs and ICIs combined with chemotherapy and anti‐angiogenesis in EGFR‐mutated NSCLC patients after EGFR‐TKI treatment failure. As expected, our results show that for those patients, combination therapy resulted in a benefit of PFS, but only ICIs+Chemo+Antiangio achieved statistical significance when compared with chemotherapy and ICIs. For patients with good performance status, ICIs+Chemo+Antiangio should be the first choice.

It is also interesting to note that the efficacy of ICIs in ORR and PFS was worse than that of chemotherapy, but OS was better than chemotherapy (although these results have no statistically significant), which may be due to the lower incidence of AEs. Making clinical decision is a complex process that requires consideration of efficacy, side effects, Karnofsky performance status, and other aspects need to be considered. This also provides a new treatment regimen for those with poor physical status who cannot tolerate chemotherapy in clinical practice.

ATLANTIC is the first prospective study to evaluate the efficacy of durvalumab as later treatment for advanced NSCLC,^57^ which was excluded because of the single‐arm study, as was the study by Jiang et al. on Toripalimab combined with chemotherapy in advanced NSCLC patients with EGFR mutation who previously treated with EGFR‐TKI.^58^

At present, an increasing number of immunotherapy‐related clinical trials are being conducted in patients with EGFR‐mutated NSCLC, and the data published so far also show good benefits and tolerable toxicity. Believe more evidence‐based medical evidence shed light for NSCLC patients with EGFR mutations who have failed EGFR‐TKI therapy.

### 4.2 Implication

This NMA offers a new therapeutic option for clinicians when making treatment decisions for NSCLC patients with EGFR mutations who have failed EGFR‐TKI therapy. ICIs+Chemo+Antiangio has been proven to be a potential optimal treatment regimen, even for BMS patients. Clinical decision making should consider that ICIs+Chemo+Antiangio can bring higher survival benefit to patients and may be the best choice. But single‐agent immune therapy can be considered when the physical condition of the patient cannot tolerate combination therapy and chemotherapy because of its low incidence of AEs and OS benefit when compared with chemotherapy. ICIs alone may not overcome the complex EGFR‐TKI resistance mechanism, so immune‐based combination therapy such as bevacizumab, similar to IMpower150, is recommended for future RCT study design. Combining with other molecularly targeted drugs may also be one direction.

### 4.3 Limitations

Our NMA has several limitations. First, in order to increase the sample size, the studies included Non‐RCT and retrospective studies in addition to RCTs, so confounding factors were inevitable. Second, although we conducted subgroup analysis based on PD‐L1 status and baseline characteristics of the population, there were few available data. We also look forward to more clinical trial data to verify the results of our subgroup analysis, and more subgroup analysis can be carried out, such as different EGFR mutation sites, etc. Third, the original objective of our study was to explore the efficacy and safety of ICIs in EGFR‐mutated NSCLC who progressed on treatment with EGFR‐TKI. When analyzing the efficacy of antiangiogenic therapy and its combination with chemotherapy, the literature is not comprehensive. Fourth, OS data from some studies is incomplete to correctly infer the effect of ICIs and combination regimens on long‐term outcomes, requiring further follow‐up, for example, the OS studied by ORIENT‐31 has not been reached so far, representing a longer OS.

## 5 CONCLUSIONS

As far as we know, this is the first NMA of the efficacy and safety of ICIs in the treatment of NSCLC patients with EGFR mutations who have failed EGFR‐TKI. Our study suggested that ICIs and their combination therapy regimens are as effective as standard chemotherapy and well tolerated, which can be used as a treatment option. Especially the ICIs+Chemo+Antiangio group may be the best choice to practice both PFS and OS. Our NMA results also need to be further confirmed in large clinical trials. Moreover, survival data from ongoing clinical trials need to be tracked to better guide clinical practice.

## AUTHOR CONTRIBUTIONS

**Zhen Wang:** Conceptualization (supporting); data curation (equal); formal analysis (equal); methodology (equal); software (equal); writing – review and editing (equal). **Fang Zhou:** Conceptualization (supporting); data curation (equal). **Shan Xu:** Formal analysis (equal). **Kang Wang:** Writing – original draft (lead). **Huan Ding:** Conceptualization (lead); formal analysis (equal); investigation (lead); methodology (equal); project administration (lead); software (equal); validation (lead).

## CONFLICT OF INTEREST STATEMENT

The authors declare no conflicts of interest.

## Supporting information


Appendix S1:

Appendix S2:

## Data Availability

The data that supports the findings of this study are available in the supplementary material of this article.
