# Supplementary material for: The efficacy and safety of immune checkpoint inhibitors for patients with EGFR‐mutated non‐small cell lung cancer who progressed on EGFR tyrosine‐kinase inhibitor therapy: A systematic review and network meta‐analysis
Source: Cancer Med. 2023 Aug 16;12(18):18516–30. doi: 10.1002/cam4.6453 (PMC10557893; doi:10.1002/cam4.6453)
Supplement: Supplementary file 1 — Appendix S1: [file CAM4-12-18516-s001.doc]

**Supplement Materials**

The efficacy and safety of immune checkpoint inhibitors for patients with EGFR-mutated non-small-cell lung cancer who progressed on EGFR tyrosine-kinase inhibitor therapy: A systematic review and network meta-analysis.

|  | Pages |
| --- | --- |
| Supplement 1: PRISMA 2020 checklist to include when reporting a systematic review involving a network meta-analysis. | 1-7 |
| Supplement 2: The searching strategy (Pubmed) | 8-12 |
| Supplement 3: The summary of the risk of bias assessment | 13-14 |
| Supplement 4: Comparisons of the fit of consistency and inconsistency | 15 |
| Supplement 5: PFS data of included studies | 16-18 |
| Supplement 6: The pooled results of PFS | 19 |
| Supplement 7: The Bayesian ranking results based on SUCRA scores | 20 |
| Supplement 8: ORR data of included studies | 21-22 |
| Supplement 9: OS data of included studies | 23-24 |
| Supplement 10: AEs data of included studies | 25-16 |
| Supplement 11: PFS data of patients with baseline brain metastases | 27 |
| Supplement 12: PFS data for patients with baseline immune status and population characteristics | 28 |
| Supplement 13: PFS data of pooled estimates of immune status in network meta-analysis | 29 |
| Supplement 14: PFS data of pooled estimates of population characteristics in network meta-analysis | 30 |
| Supplement 15: Bayesian ranking results of PFS based on SUCRA scores in subgroup analysis | 31 |


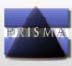
Supplement 1: PRISMA 2020 checklist to include when reporting a systematic review involving a network meta-analysis.

**PRISMA 2020 for Abstracts Checklist**

| **Section and Topic** | **Item #** | **Checklist item** | **Reported (Yes/No)** |
| --- | --- | --- | --- |
| **TITLE** | | |  |
| Title | 1 | Identify the report as a systematic review. | Yes |
| **BACKGROUND** | | |  |
| Objectives | 2 | Provide an explicit statement of the main objective(s) or question(s) the review addresses. | Yes |
| **METHODS** | | |  |
| Eligibility criteria | 3 | Specify the inclusion and exclusion criteria for the review. | Yes |
| Information sources | 4 | Specify the information sources (e.g. databases, registers) used to identify studies and the date when each was last searched. | Yes |
| Risk of bias | 5 | Specify the methods used to assess risk of bias in the included studies. | Yes |
| Synthesis of results | 6 | Specify the methods used to present and synthesise results. | Yes |
| **RESULTS** | | |  |
| Included studies | 7 | Give the total number of included studies and participants and summarise relevant characteristics of studies. | Yes |
| Synthesis of results | 8 | Present results for main outcomes, preferably indicating the number of included studies and participants for each. If meta-analysis was done, report the summary estimate and confidence/credible interval. If comparing groups, indicate the direction of the effect (i.e. which group is favoured). | Yes |
| **DISCUSSION** | | |  |
| Limitations of evidence | 9 | Provide a brief summary of the limitations of the evidence included in the review (e.g. study risk of bias, inconsistency and imprecision). | Yes |
| Interpretation | 10 | Provide a general interpretation of the results and important implications. | Yes |
| **OTHER** | | |  |
| Funding | 11 | Specify the primary source of funding for the review. | No |
| Registration | 12 | Provide the register name and registration number. | Yes |


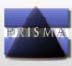
**PRISMA 2020 Checklist**

| **Section and Topic** | **Item #** | **Checklist item** | **Location where item is reported** |
| --- | --- | --- | --- |
| **TITLE** | | |  |
| Title | 1 | Identify the report as a systematic review. | 1 |
| **ABSTRACT** | | |  |
| Abstract | 2 | See the PRISMA 2020 for Abstracts checklist. | 1 |
| **INTRODUCTION** | | |  |
| Rationale | 3 | Describe the rationale for the review in the context of existing knowledge. | 2-3 |
| Objectives | 4 | Provide an explicit statement of the objective(s) or question(s) the review addresses. | 4 |
| **METHODS** | | |  |
| Eligibility criteria | 5 | Specify the inclusion and exclusion criteria for the review and how studies were grouped for the syntheses. | 5 |
| Information sources | 6 | Specify all databases, registers, websites, organisations, reference lists and other sources searched or consulted to identify studies. Specify the date when each source was last searched or consulted. | 5 |
| Search strategy | 7 | Present the full search strategies for all databases, registers and websites, including any filters and limits used. | 5 |
| Selection process | 8 | Specify the methods used to decide whether a study met the inclusion criteria of the review, including how many reviewers screened each record and each report retrieved, whether they worked independently, and if applicable, details of automation tools used in the process. | 5 |
| Data collection process | 9 | Specify the methods used to collect data from reports, including how many reviewers collected data from each report, whether they worked independently, any processes for obtaining or confirming data from study investigators, and if applicable, details of automation tools used in the process. | 5-6 |
| Data items | 10a | List and define all outcomes for which data were sought. Specify whether all results that were compatible with each outcome domain in each study were sought (e.g. for all measures, time points, analyses), and if not, the methods used to decide which results to collect. | 6 |
|  | 10b | List and define all other variables for which data were sought (e.g. participant and intervention characteristics, funding sources). Describe any assumptions made about any missing or unclear information. | 6 |
| Study risk of bias assessment | 11 | Specify the methods used to assess risk of bias in the included studies, including details of the tool(s) used, how many reviewers assessed each study and whether they worked independently, and if applicable, details of automation tools used in the process. | 6-7 |
| Effect measures | 12 | Specify for each outcome the effect measure(s) (e.g. risk ratio, mean difference) used in the synthesis or presentation of results. | 1 |
| Synthesis methods | 13a | Describe the processes used to decide which studies were eligible for each synthesis (e.g. tabulating the study intervention characteristics and comparing against the planned groups for each synthesis (item #5)). | 5 |
|  | 13b | Describe any methods required to prepare the data for presentation or synthesis, such as handling of missing summary statistics, or data conversions. | 5 |
|  | 13c | Describe any methods used to tabulate or visually display results of individual studies and syntheses. | 7 |
|  | 13d | Describe any methods used to synthesize results and provide a rationale for the choice(s). If meta-analysis was performed, describe the model(s), method(s) to identify the presence and extent of statistical heterogeneity, and software package(s) used. | 7 |
|  | 13e | Describe any methods used to explore possible causes of heterogeneity among study results (e.g. subgroup analysis, meta-regression). | 7 |
|  | 13f | Describe any sensitivity analyses conducted to assess robustness of the synthesized results. | 7 |
| Reporting bias assessment | 14 | Describe any methods used to assess risk of bias due to missing results in a synthesis (arising from reporting biases). | 6-7 |
| Certainty assessment | 15 | Describe any methods used to assess certainty (or confidence) in the body of evidence for an outcome. | No |
| **RESULTS** | | |  |
| Study selection | 16a | Describe the results of the search and selection process, from the number of records identified in the search to the number of studies included in the review, ideally using a flow diagram. | 8 |
|  | 16b | Cite studies that might appear to meet the inclusion criteria, but which were excluded, and explain why they were excluded. | 15 |
| Study characteristics | 17 | Cite each included study and present its characteristics. | 8 |
| Risk of bias in studies | 18 | Present assessments of risk of bias for each included study. | 8-9 |
| Results of individual studies | 19 | For all outcomes, present, for each study: (a) summary statistics for each group (where appropriate) and (b) an effect estimate and its precision (e.g. confidence/credible interval), ideally using structured tables or plots. | Appendix S1 5,8,9,10,11 |
| Results of syntheses | 20a | For each synthesis, briefly summarise the characteristics and risk of bias among contributing studies. | 8-9 |
|  | 20b | Present results of all statistical syntheses conducted. If meta-analysis was done, present for each the summary estimate and its precision (e.g. confidence/credible interval) and measures of statistical heterogeneity. If comparing groups, describe the direction of the effect. | 9-11 |
|  | 20c | Present results of all investigations of possible causes of heterogeneity among study results. | 9 |
|  | 20d | Present results of all sensitivity analyses conducted to assess the robustness of the synthesized results. | 12 |
| Reporting biases | 21 | Present assessments of risk of bias due to missing results (arising from reporting biases) for each synthesis assessed. | No |
| Certainty of evidence | 22 | Present assessments of certainty (or confidence) in the body of evidence for each outcome assessed. | No |
| **DISCUSSION** | | |  |
| Discussion | 23a | Provide a general interpretation of the results in the context of other evidence. | 13-15 |
|  | 23b | Discuss any limitations of the evidence included in the review. | 16 |
|  | 23c | Discuss any limitations of the review processes used. | 16 |
|  | 23d | Discuss implications of the results for practice, policy, and future research. | 15-16 |
| **OTHER INFORMATION** | | |  |
| Registration and protocol | 24a | Provide registration information for the review, including register name and registration number, or state that the review was not registered. | 4 |
|  | 24b | Indicate where the review protocol can be accessed, or state that a protocol was not prepared. | 4 |
|  | 24c | Describe and explain any amendments to information provided at registration or in the protocol. | No |
| Support | 25 | Describe sources of financial or non-financial support for the review, and the role of the funders or sponsors in the review. | No |
| Competing interests | 26 | Declare any competing interests of review authors. | 17 |
| Availability of data, code and other materials | 27 | Report which of the following are publicly available and where they can be found: template data collection forms; data extracted from included studies; data used for all analyses; analytic code; any other materials used in the review. | Appendix S1 and S2 for Review |

Supplement 2: The searching strategy

Pubmed

((((("Lung Neoplasms"[Mesh]) OR (((((((((((((((((Pulmonary Neoplasms[Title/Abstract]) OR (Neoplasms, Lung[Title/Abstract])) OR (Lung Neoplasm[Title/Abstract])) OR (Neoplasm, Lung[Title/Abstract])) OR (Neoplasms, Pulmonary[Title/Abstract])) OR (Neoplasm, Pulmonary[Title/Abstract])) OR (Pulmonary Neoplasm[Title/Abstract])) OR (Lung Cancer[Title/Abstract])) OR (Cancer, Lung[Title/Abstract])) OR (Cancers, Lung[Title/Abstract])) OR (Lung Cancers[Title/Abstract])) OR (Pulmonary Cancer[Title/Abstract])) OR (Cancer, Pulmonary[Title/Abstract])) OR (Cancers, Pulmonary[Title/Abstract])) OR (Pulmonary Cancers[Title/Abstract])) OR (Cancer of the Lung[Title/Abstract])) OR (Cancer of Lung[Title/Abstract]))) OR (("Carcinoma, Non-Small-Cell Lung"[Mesh]) OR (((((((((((Carcinoma, Non Small Cell Lung[Title/Abstract]) OR (Carcinomas, Non-Small-Cell Lung[Title/Abstract])) OR (Lung Carcinoma, Non-Small-Cell[Title/Abstract])) OR (Lung Carcinomas, Non-Small-Cell[Title/Abstract])) OR (Non-Small-Cell Lung Carcinomas[Title/Abstract])) OR (Non-Small Cell Lung Carcinoma[Title/Abstract])) OR (Non-Small-Cell Lung Carcinoma[Title/Abstract])) OR (Non Small Cell Lung Carcinoma[Title/Abstract])) OR (Nonsmall Cell Lung Cancer[Title/Abstract])) OR (Carcinoma, Non-Small Cell Lung[Title/Abstract])) OR (Non-Small Cell Lung Cancer[Title/Abstract])))) AND (("ErbB Receptors"[Mesh]) OR ((((((((((((((((((((((((((((((((((((((((((((((((((((((((ErbB Receptor[Title/Abstract]) OR (Receptor, ErbB[Title/Abstract])) OR (Transforming Growth Factor alpha Receptor[Title/Abstract])) OR (Receptor, TGF-alpha[Title/Abstract])) OR (Receptor, TGF alpha[Title/Abstract])) OR (TGF-alpha Receptor[Title/Abstract])) OR (Epidermal Growth Factor Receptor Kinase[Title/Abstract])) OR (Epidermal Growth Factor Receptor Protein-Tyrosine Kinase[Title/Abstract])) OR (Epidermal Growth Factor Receptor Protein Tyrosine Kinase[Title/Abstract])) OR (Receptor, Urogastrone[Title/Abstract])) OR (Receptor, Transforming-Growth Factor alpha[Title/Abstract])) OR (Receptor, Transforming Growth Factor alpha[Title/Abstract])) OR (Urogastrone Receptor[Title/Abstract])) OR (HER Family Receptors[Title/Abstract])) OR (Family Receptors, HER[Title/Abstract])) OR (Receptors, HER Family[Title/Abstract])) OR (HER Family Receptor[Title/Abstract])) OR (Family Receptor, HER[Title/Abstract])) OR (Receptor, HER Family[Title/Abstract])) OR (Receptor, ErbB-1[Title/Abstract])) OR (ErbB-1 Receptor[Title/Abstract])) OR (Receptor, ErbB 1[Title/Abstract])) OR (Proto-oncogene c-ErbB-1 Protein[Title/Abstract])) OR (Proto oncogene c ErbB 1 Protein[Title/Abstract])) OR (c-ErbB-1 Protein, Proto-oncogene[Title/Abstract])) OR (c-erbB-1 Protein[Title/Abstract])) OR (c erbB 1 Protein[Title/Abstract])) OR (Receptor Tyrosine-protein Kinase erbB-1[Title/Abstract])) OR (Receptor Tyrosine protein Kinase erbB 1[Title/Abstract])) OR (erbB-1 Proto-Oncogene Protein[Title/Abstract])) OR (Proto-Oncogene Protein, erbB-1[Title/Abstract])) OR (erbB 1 Proto Oncogene Protein[Title/Abstract])) OR (EGF Receptors[Title/Abstract])) OR (Receptors, EGF[Title/Abstract])) OR (Epidermal Growth Factor Receptor[Title/Abstract])) OR (Receptors, Epidermal Growth Factor-Urogastrone[Title/Abstract])) OR (Receptors, Epidermal Growth Factor Urogastrone[Title/Abstract])) OR (Epidermal Growth Factor Receptor Family Protein[Title/Abstract])) OR (Receptor, EGF[Title/Abstract])) OR (Receptors, Epidermal Growth Factor[Title/Abstract])) OR (Epidermal Growth Factor Receptor Family Proteins[Title/Abstract])) OR (EGF Receptor[Title/Abstract])) OR (Receptor, Epidermal Growth Factor[Title/Abstract])) OR (EGFR[Title/Abstract])) OR (Gefitinib Iressa[Title/Abstract])) OR (Erlotinib[Title/Abstract])) OR (CP-358774[Title/Abstract])) OR (Tarceva[Title/Abstract])) OR (Ectinib[Title/Abstract])) OR (Afatinib[Title/Abstract])) OR (Dactinib[Title/Abstract])) OR (T790M[Title/Abstract])) OR (Ositinib[Title/Abstract])) OR (AZD9291[Title/Abstract])) OR (Ametinib[Title/Abstract])) OR (Furmonertinib[Title/Abstract])))) AND ((((((((((("Nivolumab"[Mesh]) OR ((((((((((Opdivo[Title/Abstract]) OR (ONO-4538[Title/Abstract])) OR (ONO 4538[Title/Abstract])) OR (ONO4538[Title/Abstract])) OR (MDX-1106[Title/Abstract])) OR (MDX 1106[Title/Abstract])) OR (MDX1106[Title/Abstract])) OR (BMS-936558[Title/Abstract])) OR (BMS 936558[Title/Abstract])) OR (BMS936558[Title/Abstract]))) OR ("Ipilimumab"[Mesh])) OR (((((((((Anti-CTLA-4 MAb Ipilimumab) OR (Anti CTLA 4 MAb Ipilimumab)) OR (Ipilimumab, Anti-CTLA-4 MAb)) OR (Yervoy)) OR (MDX 010)) OR (MDX010)) OR (MDX-010)) OR (MDX-CTLA-4)) OR (MDX CTLA 4))) OR ("Antibodies, Monoclonal"[Mesh])) OR (((Monoclonal Antibodies[Title/Abstract]) OR (Monoclonal Antibody[Title/Abstract])) OR (Antibody, Monoclonal[Title/Abstract]))) OR ("Immunoglobulins"[Mesh])) OR (((((Globulins, Immune[Title/Abstract]) OR (Immunoglobulin[Title/Abstract])) OR (Immune Globulin[Title/Abstract])) OR (Globulin, Immune[Title/Abstract])) OR (Immune Globulins[Title/Abstract]))) OR ("Immunologic Factors"[Mesh])) OR (((((((((((((((((((((((((((((((((((((((((((((((((((((((Immunological Factors[Title/Abstract]) OR (Factors, Immunological[Title/Abstract])) OR (Factors, Immunologic[Title/Abstract])) OR (Immune Factors[Title/Abstract])) OR (Factors, Immune[Title/Abstract])) OR (Immune Factor[Title/Abstract])) OR (Factor, Immune[Title/Abstract])) OR (Immunological Factor[Title/Abstract])) OR (Factor, Immunological[Title/Abstract])) OR (Immunomodulators[Title/Abstract])) OR (Immunomodulator[Title/Abstract])) OR (Biological Response Modifiers[Title/Abstract])) OR (Biological Response Modifier[Title/Abstract])) OR (Modifier, Biological Response[Title/Abstract])) OR (Response Modifier, Biological[Title/Abstract])) OR (Biomodulators[Title/Abstract])) OR (Response Modifiers, Biological[Title/Abstract])) OR (Biomodulator[Title/Abstract])) OR (Modifiers, Biological Response[Title/Abstract])) OR (Pembrolizumab[Title/Abstract])) OR (Keytruda[Title/Abstract])) OR (MK-3475[Title/Abstract])) OR (Durvalumab[Title/Abstract])) OR (Imfinzi[Title/Abstract])) OR (MEDI 4736[Title/Abstract])) OR (Atezolizumab[Title/Abstract])) OR (Tecentrip[Title/Abstract])) OR (MPDL3280A[Title/Abstract])) OR (avelumab[Title/Abstract])) OR (Bavencio[Title/Abstract])) OR (MSB 0010718C[Title/Abstract])) OR (MSB0010718C[Title/Abstract])) OR (tremelimumab[Title/Abstract])) OR (checkpointinhibitors[Title/Abstract])) OR (Lambrolizumab[Title/Abstract])) OR (Programmed Cell Death Ligand 1/PD-1[Title/Abstract])) OR (Programmed cell death 1 ligand 1/PD-L1[Title/Abstract])) OR (Sintilimab[Title/Abstract])) OR (IBI308[Title/Abstract])) OR (Camrelizumab[Title/Abstract])) OR (SHR-1210[Title/Abstract])) OR (Tislelizumab[Title/Abstract])) OR (BGB-A317[Title/Abstract])) OR (Toripalimab[Title/Abstract])) OR (JS001[Title/Abstract])) OR (serplulimab[Title/Abstract])) OR (HLX10[Title/Abstract])) OR (CemiplimabLibtayo[Title/Abstract])) OR (TremelimumabImjudo[Title/Abstract])) OR (CTLA-4[Title/Abstract])) OR (SugemalimabCejemly[Title/Abstract])) OR (Immunotherapy[Title/Abstract])) OR (Immunotherapies[Title/Abstract])) OR (PD-1[Title/Abstract])) OR (PD-L1[Title/Abstract]))) OR (("Antineoplastic Agents, Immunological"[Mesh]) OR ((((((((((Immunological Antineoplastic Agents[Title/Abstract]) OR (Antineoplastics, Monoclonal Antibodies[Title/Abstract])) OR (Antibodies Antineoplastics, Monoclonal[Title/Abstract])) OR (Monoclonal Antibodies Antineoplastics[Title/Abstract])) OR (Monoclonal Antibodies, Antineoplastic[Title/Abstract])) OR (Antineoplastic Monoclonal Antibodies[Title/Abstract])) OR (Antineoplastic MAbs[Title/Abstract])) OR (Antineoplastics, Monoclonal Antibody[Title/Abstract])) OR (Monoclonal Antibody Antineoplastics[Title/Abstract])) OR (MAbs, Antineoplastic[Title/Abstract]))))) AND (((("Disease Progression"[Mesh]) OR ((((((Progression, Disease[Title/Abstract]) OR (Clinical Course[Title/Abstract])) OR (Clinical Progression[Title/Abstract])) OR (Progression, Clinical[Title/Abstract])) OR (Disease Exacerbation[Title/Abstract])) OR (Exacerbation, Disease[Title/Abstract]))) OR ("Drug Resistance, Neoplasm"[Mesh])) OR (((((((((((((Resistance, Antineoplastic Agent[Title/Abstract]) OR (Resistance, Antineoplastic Drug[Title/Abstract])) OR (Drug Resistance, Antineoplastic[Title/Abstract])) OR (Neoplasm Drug Resistance[Title/Abstract])) OR (Antineoplastic Agent Resistance[Title/Abstract])) OR (Antineoplastic Drug Resistance[Title/Abstract])) OR (Antibiotic Resistance, Neoplasm[Title/Abstract])) OR (Disease Progressions[Title/Abstract])) OR (Progression, Disease[Title/Abstract])) OR (Progressions, Disease[Title/Abstract])) OR (Disease Exacerbation[Title/Abstract])) OR (Disease Progression[Title/Abstract])) OR (progressed[Title/Abstract])))

**Supplement 3: The summary of the risk of bias assessment**

| **A** | 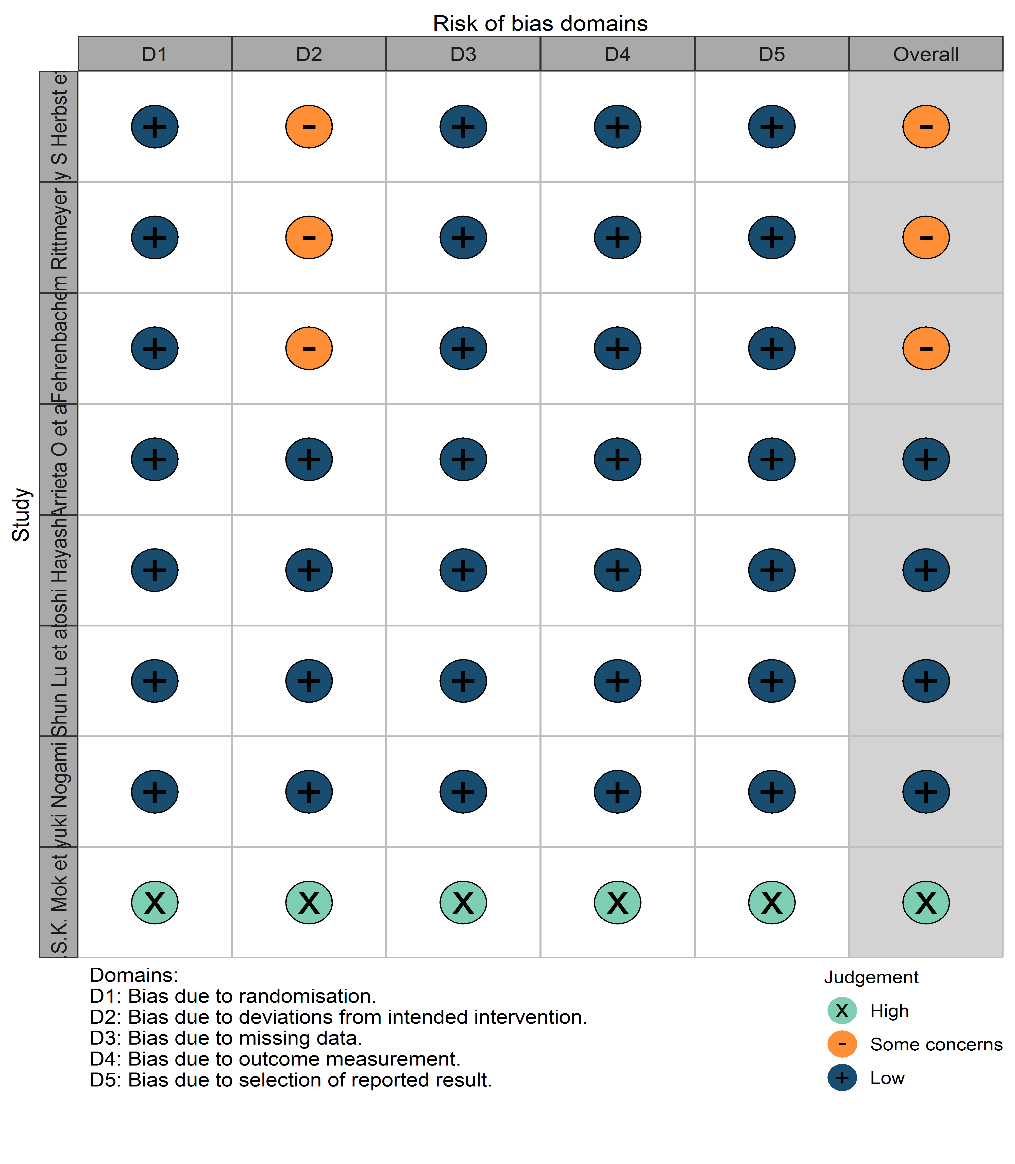 | **B** | 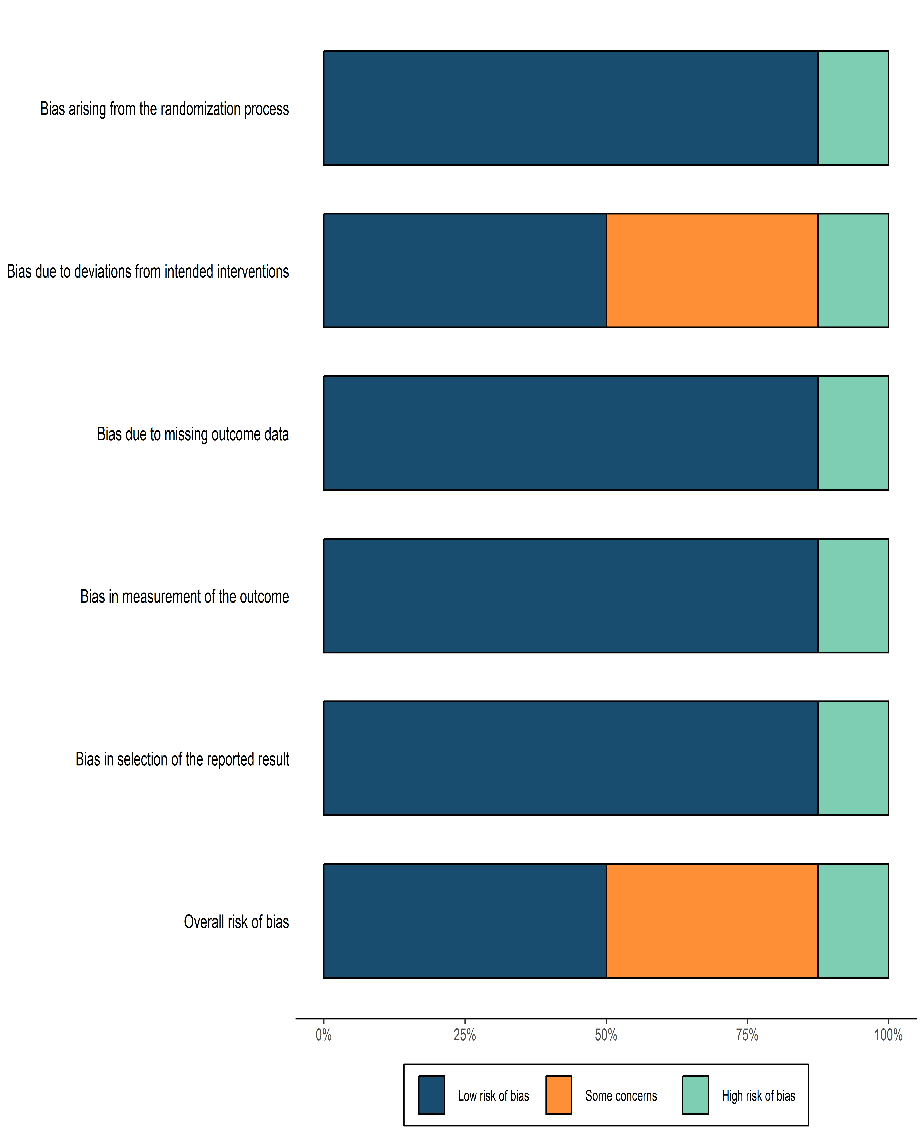 |
| --- | --- | --- | --- |
| **C** | 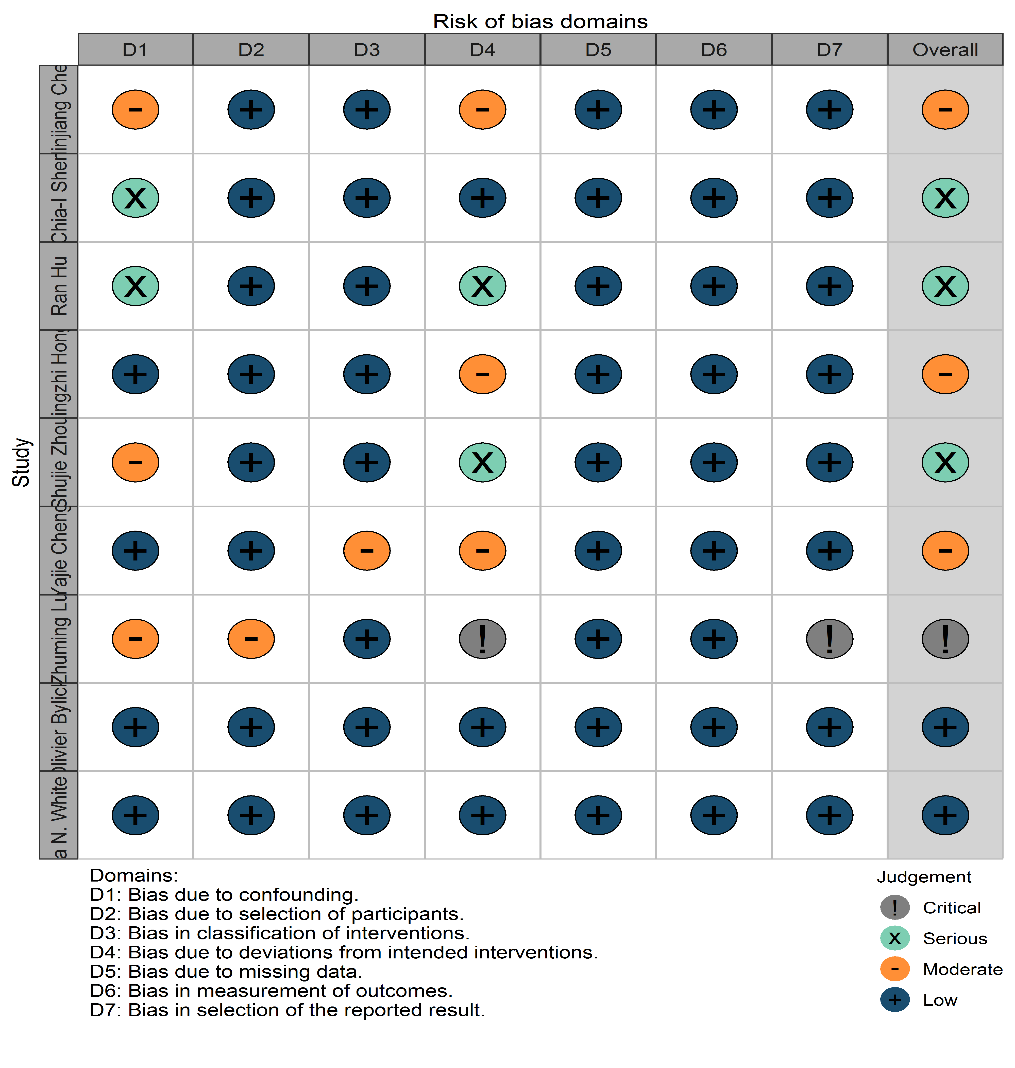 | **D** | 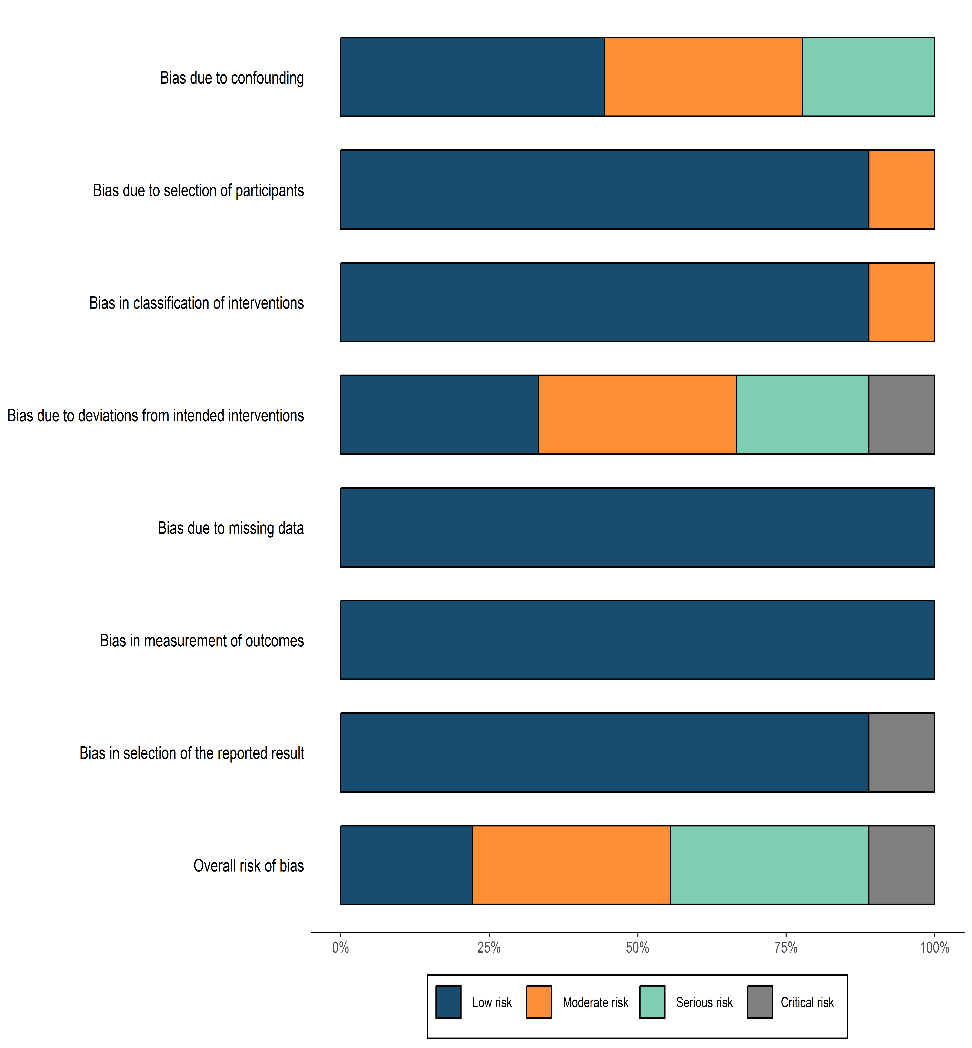 |

**A** Traffic_light diagram of the RCTs  **B** Summary of bias risk assessment for RCTs  **C** Summary of bias risk assessment for Non-RCTs and retrospective studies  **D** Summary of bias risk assessment for Non-RCTs and retrospective studies

Supplement 4: Comparisons of the fit of consistency and inconsistency

|  | **Overall** | | | | | | **Brain metastases** |
| --- | --- | --- | --- | --- | --- | --- | --- |
|  |  |  |  |  |  |  | **at baseline** |
| **Model** | PFS | OS | ORR | AEs | | | PFS |
|  |  |  |  | Any grade | Grade≥3 AEs | Leading to treatment discontinuation occurred |  |
| **Consistency** | 15.93 | 9.13 | 17.38 | 11.43 | 20.53 | 15.37 | 3.9 |
| **Inconsistency** | 16.19 | 9.12 | 17.39 | 11.48 | 20.56 | 15.31 | 4.0 |

Supplement 5. PFS data of included studies

| Study (Author, year) | Study type | Intervention vs. Control | HR ((95% CI) |
| --- | --- | --- | --- |
| Roy S Herbst et al, 2015 | RCT | Pembrolizumab vs. Docetaxel | 1.79 0.94–3.42) |
| Achim Rittmeyer et al, 2016 | RCT | Atezolizumab vs. Docetaxel | 0.95 (0.82-1.10) |
| Arrieta O et al, 2020 | RCT | Pembrolizumab+Docetaxel vs. Docetaxel | 0.26 (0.07-92.60） |
| Hidetoshi Hayashi et al, 2022 | RCT | Nivolumab vs. Carboplatin–pemetrexed | 1.92 (1.27-2.90) |
| Shun Lu et al, 2022 | RCT | Sintilimab+IBI305+pemetrexed+cisplatin vs. pemetrexed+cisplatin | 0.46 (0.34-0.64） |
|  |  | Sintilimab+pemetrexed+cisplatin vs. pemetrexed+cisplatin | 0.72 (0.552-0.948) |
| Naoyuki Nogami et al, 2022 | RCT | Atezolizumab+bevacizumab+carboplatin/paclitaxel vs. Bevacizumab+carboplatin/paclitaxel | 0.42 (0.22-0.8) |
|  |  | Atezolizumab+carboplatin/paclitaxel vs. Bevacizumab+carboplatin/paclitaxel | 1.24 (0.72-2.15) |
| T.S.K. Mok et al, 2022 | RCT | Nivolumab +Pemetrexed+Cisplatin/Carboplatin vs. Pemetrexed+Cisplatin/Carboplatin | 0.75 (0.56-1.00) |
| Chia‑I Shen et al, 2021 | Retrospective study | ICI+Chemotherapy vs. ICI | 0.24 (0.06-0.97) |
| Ran Hu et al, 2021 | Retrospective study | PD-1 inhibitors+ Chemotherapy vs.PD-1 inhibitors | 0.69 (0.31-1.57)^#^ |
|  |  | PD-1 inhibitors+Bevacizumab vs.PD-1 inhibitors | 0.65 (0.22-1.95)^#^ |
| Lingzhi Hong et al, 2022 | Retrospective study | Chemo+Bev+IO vs. Chemotherapy | 0.85 (0.36-1.96)^*^ |
|  |  | ChemoIO VS... Chemotherapy | 0.67 (0.4-1.14)^*^ |
|  |  | IO-mono vs.Chemotherapy | 2.22 (1.37-3.59) |
|  |  | Chemo+Bev vs.Chemotherapy | 0.78 (0.46-1.31)^*^ |
| Zhuming Lu et al, 2022 | Retrospective study | Pembrolizumab vs.Pemetrexed + platinum | 0.25 (0.15-0.55) ^#^ |
| Shujie Zhou et al, 2022 | Retrospective study | ICI + Chemotherapy + Antiangiogenic vs.ICI monotherapy | 0.13 (0.04-0.5)^*^ |
|  |  | ICI + Chemotherapy vs. ICI monotherapy | 0.15 (0.04-0.57)^*^ |
|  |  | ICI + Antiangiogenic vs.ICI monotherapy | 0.26 (0.07-1.04)^*^ |
|  |  | ICI + Chemotherapy vs. Chemotherapy | 0.71 (0.21-2.79) ^#^ |
|  |  | ICI+Chemotherapy vs.Chemotherapy vs. ICI | 0.24 (0.06-0.97) ^*^ |
| Yajie Cheng etal, 2022 | Retrospective study | ICI+Chemotherapy vs. Chemotherapy | 0.71 (0.48-1.05) |
| Olivier Bylicki et al, 2023 | Non-RCT | ICIs+Chemotherapy+Bev vs.ICIs+Chemotherapy | 0.88 (0.58-1.35)^#^ |
| Minjiang Chen et al, 2023 | Retrospective study | Pembrolizumab+ Chemotherapy vs. Chemotherapy | 0.64 (0.46-0.9） |

Bev: Bevacizumab Chemo: Chemotherapy IO: Immunotherapy

* Adjusted data

^#^ Data extracted from the survival curve

Supplement 6: The combined results of PFS and OS **(**combined HRs (95% credible intervals) for OS in the upper triangle and PFS in the lower triangle**)**

Supplement 7: The Bayesian ranking results based on SUCRA scores

| Treatment | Ranking first of possibility (%) | | | | | | |
| --- | --- | --- | --- | --- | --- | --- | --- |
|  | PFS | OS | ORR | AEs | | | Brain metastases at baseline |
|  |  |  |  | Any grade | Grade≥3 AEs | Leading to treatment discontinuation occurred | Pooled PFS |
| ICIs+Chemo | 56.8 | 51.0 | 66.9 | 31.5 | 62.6 | 52.0 | 58.0 |
| Chemo | 33.5 | 18.8 | 37.3 | 53.0 | 39.9 | 40.9 | 51.2 |
| ICIs | 8.3 | 23.8 | 5.1 | 1.0 | 31.9 | 6.2 | 7.0 |
| ICIs+Chemo+Antiangio | 95.9 | 90.1 | 90.7 | 64.8 | 50.7 | 98.8 | 88.4 |
| Antiangio+Chemo | 55.5 | 66.3 | / | 99.7 | 64.9 | 52.1 | / |
| ICIs+Antiangio | / | / | / | / | / | / | 45.6 |

Supplement 8: ORR data of included studies

| Study (Author, year) | Study type | Intervention vs. Control | Responders/Sample size |
| --- | --- | --- | --- |
| Arrieta O et al, 2020 | RCT | Pembrolizumab+Docetaxel | 7/12 |
|  |  | Docetaxel | 3/13 |
| Hidetoshi Hayashi et al, 2022 | RCT | Nivolumab | 5/52 |
|  |  | Carboplatin–pemetrexed | 18/50 |
| Shun Lu et al, 2022 | RCT | Sintilimab+IBI305+pemetrexed+cisplatin | 71/148 |
|  |  | Sintilimab+pemetrexed+cisplatin | 50/145 |
|  |  | Pemetrexed+cisplatin | 44/151 |
| T.S.K. Mok et al, 2022 | RCT | Nivolumab +Pemetrexed+Cisplatin/Carboplatin | 45/144 |
|  |  | Pemetrexed+Cisplatin/Carboplatin | 41/150 |
| Ran Hu et al, 2021 | Retrospective study | PD-1 inhibitors+ Chemotherapy | 4/21 |
|  |  | PD-1 inhibitors + Chemotherapy+ Bevacizumab | 0/20 |
|  |  | PD-1 inhibitors+Bevacizumab | 2/8 |
|  |  | PD-1 inhibitors | 0/7 |
| Zhuming Lu et al, 2022 | Retrospective study | Pembrolizumab | 5/32 |
|  |  | Pemetrexed + platinum | 3/32 |
| Shujie Zhou et al, 2022 | Retrospective study | ICI + Chemotherapy + Antiangiogenic | 4/18 |
|  |  | ICI + Chemotherapy | 7/19 |
|  |  | ICI + Antiangiogenic | 0/12 |
|  |  | ICI monotherapy | 0/4 |
| Minjiang Chen et al, 2023 | Retrospective study | Pembrolizumab+ Chemotherapy | 28/82 |
|  |  | Chemotherapy | 17/82 |

Supplement 9: OS data of included studies

| Study (Author, year) | Study type | Intervention vs. Control | HR (95% CI) |
| --- | --- | --- | --- |
| Roy S Herbst et al, 2015 | RCT | Pembrolizumab vs. Docetaxel | 0.88 (0.45–1.70) |
| Louis Fehrenbacher et al, 2016 | RCT | Atezolizumab vs. Docetaxel | 0.99 (0.29–3.40) |
| Achim Rittmeyer et al, 2016 | RCT | Atezolizumab vs. Docetaxel | 1.24(0.71-2.18) |
| Arrieta O et al, 2020 | RCT | Pembrolizumab+Docetaxel vs. Docetaxel | 1.05(0.31-3.55) |
| Hidetoshi Hayashi et al, 2022 | RCT | Nivolumab vs. Carboplatin–pemetrexed | 0.88(0.53-1.47) |
| Naoyuki Nogami et al, 2022 | RCT | Atezolizumab+bevacizumab+carboplatin/paclitaxel vs. Bevacizumab+carboplatin/paclitaxel | 0.74(0.38-1.46) |
|  |  | Atezolizumab+carboplatin/paclitaxel vs. Bevacizumab+carboplatin/paclitaxel | 1.22(0.68-2.22) |
| T.S.K. Mok et al, 2022 | RCT | Nivolumab +Pemetrexed+Cisplatin/Carboplatin vs. Pemetrexed+Cisplatin/Carboplatin | 0.82 (0.61-1.10) |
| Chia‑I Shen et al, 2021 | Retrospective study | ICI+Chemotherapy vs. ICI | 0.53（0.05-5.32)^*^ |
| Maya N. White et al, 2021 | Retrospective study | Chemo-IO vs. Chemotherapy | 2.66（1.25-5.65)^*^ |
|  |  | Chemo-Bev vs. Chemotherapy | 1.5（0.84-2.69)^*^ |
| Lingzhi Hong et al, 2022 | Retrospective study | Chemo+Bev+IO vs. Chemotherapy | 1.57(0.57-4.3)^*^ |
|  |  | ChemoIO vs. Chemotherapy | 1.1(0.59-2.05)^*^ |
|  |  | IO-mono vs. Chemotherapy | 1.63(0.95-2.79) |
|  |  | Chemo+Bev vs. Chemotherapy | 1.14(0.65-1.99)^*^ |
| Yajie Cheng etal, 2022 | Retrospective study | ICI+Chemotherapy vs. Chemotherapy | 0.58(0.37-0.91) |
| Olivier Bylicki et al, 2023 | Non-RCT | ICIs+Chemotherapy+Bev vs. ICIs+Chemotherapy | 0.91（0.45-1.85)^#^ |
| Minjiang Chen et al, 2023 | Retrospective study | Pembrolizumab+ Chemotherapy vs. Chemotherapy | 0.49 (0.32-0.75) |

* Adjusted data

^#^ Data extracted from the survival curve

Supplement 10: AEs data of included studies

| Study (Author, year) | Study type | Groups | AEs of any-grade | AEs of grade greater than or equal to 3 | AEs leading to treatment discontinuation occurred |
| --- | --- | --- | --- | --- | --- |
|  |  |  | Responders/Sample size | | |
| Arrieta O et al, 2020 | RCT | Pembrolizumab +Docetaxel | NG | 0/12 | 0/12 |
|  |  | Docetaxel | NG | 0/13 | 0/13 |
| Hidetoshi Hayashi et al, 2022 | RCT | Nivolumab | 32/52 | 5/52 | 3/52 |
|  |  | carboplatin–pemetrexed | 41/50 | 6/50 | 7/50 |
| Shun Lu et al, 2022 | RCT | Sintilimab+IBI305+pemetrexed+cisplatin | 145/148 | 81/148 | 25/148 |
|  |  | Sintilimab+pemetrexed+cisplatin | 139/145 | 57/145 | 12/145 |
|  |  | pemetrexed+cisplatin | 147/151 | 77/151 | 10/151 |
| Naoyuki Nogami et al, 2022 | RCT | atezolizumab+bevacizumab+carboplatin/paclitaxel | 22/22 | 10/22 | 9/22 |
|  |  | atezolizumab+carboplatin/paclitaxel | 27/28 | 20/28 | 4/28 |
|  |  | bevacizumab+carboplatin/paclitaxel | 28/28 | 18/28 | 5/28 |
| T.S.K. Mok et al, 2022 | RCT | Nivolumab +Pemetrexed+Cisplatin/Carboplatin | NG | 65/144 | NG |
|  |  | Pemetrexed+Cisplatin/Carboplatin | NG | 44/150 | NG |
| Minjiang Chen et al, 2023 | Retrospectiv study | Pembrolizumab+ chemotherapy | NG | 37/82 | NG |
|  |  | chemotherapy | NG | 35/82 | NG |
| Zhuming Lu et al, 2022 | Retrospectiv study | Pembrolizumab | 9/32 | 0/32 | NG |
|  |  | pemetrexed plus platinum | 13/32 | 2/32 | NG |
| Yajie Cheng et al, 2022 | Retrospectiv study | ICI+chemotherapy | NG | NG | 1/61 |
|  |  | chemotherapy | NG | NG | 0/60 |

Supplement 11: PFS data of patients with baseline brain metastases

| Study (Author, year) | Study type | Intervention vs. Control | HR ((95% CI) |
| --- | --- | --- | --- |
| Hidetoshi Hayashi et al, 2022 | RCT | Nivolumab vs. Carboplatin–pemetrexed | 3.7 (1.6-8.59) |
| Shun Lu et al, 2022 | RCT | Sintilimab+IBI305+pemetrexed+cisplatin vs. pemetrexed+cisplatin | 0.48 (0.29-0.79） |
| Chia‑I Shen et al, 2021 | Retrospective study | ICI+Chemotherapy vs. ICI | 1.2 (0.41-3.55) |
| Shujie Zhou et al, 2022 | Retrospective study | ICI + Chemotherapy + Antiangiogenic vs.ICI monotherapy | 0.13 (0.04-0.5)^*^ |
|  |  | ICI + Chemotherapy vs. ICI monotherapy | 0.15 (0.04-0.57)^*^ |
|  |  | ICI + Antiangiogenic vs.ICI monotherapy | 0.26 (0.07-1.04)^*^ |
|  |  | ICI + Chemotherapy vs. Chemotherapy | 0.71 (0.21-2.79) ^#^ |
|  |  | ICI+Chemotherapy vs.Chemotherapy vs. ICI | 0.24 (0.06-0.97) ^*^ |
| Minjiang Chen et al, 2023 | Retrospective study | Pembrolizumab+ Chemotherapy vs. Chemotherapy | 0.68 (0.36-1.26） |

* Adjusted data

^#^ Data extracted from the survival curve

Supplement 12: PFS data for patients with baseline immune status and population characteristics

| Study (Author, year) | Study type | Intervention vs. Control | HR ((95% CI) | | | | | | |
| --- | --- | --- | --- | --- | --- | --- | --- | --- | --- |
|  |  |  | Baseline immune status | | | Gender | | Tobacco use history | |
|  |  |  | PD-L1 ＜1% | PD-L1 1 - 49% | PD-L1 >50% | Male | Female | Never smoked | Current or former smoker |
| Hidetoshi Hayashi et al, 2022 | RCT | Nivolumab vs. Carboplatin–pemetrexed | 1.67（0.9-3.1） | 2.1（0.83-5.29） | 1.49（0.31-7.24） | 1.92（1.01-3.67） | 1.69（0.99-2.87） | 1.79（1.04-3.07 | 1.70（0.92-3.13 |
| Shun Lu et al, 2022 | RCT | Sintilimab+IBI305+pemetrexed+cisplatin vs. pemetrexed+cisplatin | NG | NG | NG | 0.67（0.41-1.09） | 0.44（0.3-0.66） | 0.5（0.35-0.71） | 0.62（0.35-1.1） |
| T.S.K. Mok et al, 2022 | RCT | ICI+Chemotherapy vs. ICI | 0.91 (0.58-1.44) | 0.88 (0.52-1.51) | 0.65 (0.36-1.15) | NG | NG | NG | NG |
| Minjiang Chen et al, 2023 | Retrospective study | Pembrolizumab+ Chemotherapy vs. Chemotherapy | NG | NG | NG | 0.7859（0.4734-1.305） | 0.5260（0.3417-0.8096） | 0.6218（0.3008-1.285） | 0.6482（0.4487-0.9363） |

Supplement 13: PFS data of baseline immune status subgroup analysis

Supplement 14: PFS data of population characteristics subgroup analysis

Supplement 15: Bayesian ranking results of PFS based on SUCRA scores in subgroup analysis

| Treatment | Ranking first of possibility (%) | | | |
| --- | --- | --- | --- | --- |
|  | Gender | | Tobacco use history | |
|  | Male | Female | Never smoked | Current or former smoker |
| ICIs+Chemo | 67.6 | 74.2 | 70.3 | 76.1 |
| Chemo | 45.1 | 33.4 | 36.3 | 38.9 |
| ICIs | 7.7 | 8.0 | 6.7 | 7.1 |
| ICIs+Chemo+Antiangio | 79.6 | 84.4 | 87.0 | 77.9 |
