# Supplementary material for: The efficacy and safety of immune checkpoint inhibitors for patients with EGFR‐mutated non‐small cell lung cancer who progressed on EGFR tyrosine‐kinase inhibitor therapy: A systematic review and network meta‐analysis
Source: Cancer Med. 2023 Aug 16;12(18):18516–30. doi: 10.1002/cam4.6453 (PMC10557893; doi:10.1002/cam4.6453)
Supplement: Supplementary file 2 — Appendix S2: [file CAM4-12-18516-s002.docx]

R language code for PFS:

1.setwd("C:\\Users\\Administrator\\Desktop\\PFS")

2. library("gemtc")

library("rjags")

library(ggplot2)

3. data1 <- read.table("data1.txt", sep="\t", header=T, check.names=F)

4.treatments <- read.table(textConnection(

'id description

1 "ICIs+Chemo"

2 "Chemo"

3 "ICIs"

4 "ICIs+Chemo+Antiangio"

5 "Antiangio+Chemo"

'), header=TRUE)

5.network <- mtc.network(data.re=data1,description="PFS network", treatments=treatments)

summary(network)

plot(network, use.description = TRUE, vertex.label.cex=1.5, vertex.size=20, vertex.shape="circle", vertex.label.color="darkblue", vertex.label.dist=3, vertex.label.degree=-pi/3, vertex.color="blue", dynamic.edge.width=TRUE,edge.color="gray", vertex.label.font=2)

tiff("network.tiff", height=5000,width=5000, res= 600)

plot(network, use.description = TRUE, vertex.label.cex=1.5, vertex.size=20, vertex.shape="circle", vertex.label.color="darkblue", vertex.label.dist=3, vertex.label.degree=-pi/3, vertex.color="blue", dynamic.edge.width=TRUE,edge.color="gray", vertex.label.font=2)

dev.off()

6.model.ran <- mtc.model(network, likelihood="binom", link="cloglog", type = "consistency", linearModel='random',dic=TRUE)

cat(model.ran$code)

result.ran <- mtc.run(model.ran, n.adapt=10000, n.iter=200000, thin=10)

gelman.diag(result.ran)

7.gelman.plot(result.ran)

tiff("dcslzd.tiff", height=5000,width=5000, res= 600)

gelman.plot(result.ran, digits = 2)

dev.off()

8.summary(result.ran)

9.plot(result.ran)

9. tiff("dcgjmdt.tiff", height=5000,width=4000, res= 600)

plot(result.ran, digits = 2)

dev.off()

10. forest(relative.effect(result.ran, t1="2"), use.description = TRUE)

tiff("senlintu.tiff", height=5000,width=4000, res= 600)

p1<- forest(relative.effect(result.ran, t1="2"), use.description = TRUE)

p1

dev.off()

11. a <- round(exp(relative.effect.table(result.ran)),2)

write.csv(a, "PFS-network-weixin.csv")

12.modelume <-mtc.model(network, type="ume", n.chain=4, likelihood="binom", link="cloglog", linearModel="random")

resultsume <- mtc.run(modelume, n.adapt = 10000, n.iter = 200000, thin = 10)

summary(resultsume)

pdf(file="jiedianpilie2.pdf",width = 10,height = 7)

plot(b)

dev.off()

13.resultanohe <- mtc.anohe(network, n.adapt = 5000, n.iter = 100000, thin = 10, n.chain=10, likelihood="binom", link="cloglog",linearModel="random")

c<-summary(resultanohe)

plot(c)

pdf(file="yizhixingjianyanweixin.pdf",width = 10,height = 7)

plot(c)

dev.off()

14. ranks <- rank.probability(result.ran,preferredDirection=-1)

print(ranks)

write.csv(ranks, "rank.csv")

15.tiff("nma.rank.tiff", height=5000,width=4000, res= 600)

plot(ranks,xlab='treatment', col=c("lightblue", "mistyrose", "lightcyan", "lavender"), ylab='Cumulative probability')

dev.off()

16. sucraranks <-sucra(ranks)

print(sucraranks)

plot(sucraranks)

17. data1rank <- read.table(file="data3.txt",header=TRUE)

data1rank

p<-ggplot(data1rank, aes(x=rank, y=prob, colour=treatments)) +geom_line(linewidth=2, lty="solid")

p1<-p+geom_point(size=5)+labs(x="Rank", y="Probability")+theme(axis.text = element_text(size=25),axis.title=element_text(size=28),panel.background=element_blank(),axis.line = element_line(color="black"), legend.title=element_text(size=35, color="black"),legend.position="bottom")+theme(legend.title = element_blank())+scale_color_manual(values = c('#ec1c24','#fdbd10','#0066b2','#9933CC','#FF00CC'))+theme(legend.text=element_text(family = "serif", size=20))

p1

tiff("nma.rank.tiff", height=5000,width=7000, res= 600)

p1

dev.off()

R language code for ORR:

1.setwd("C:\\Users\\Administrator\\Desktop\\ ORR")

2. library("gemtc")

library("rjags")

library(ggplot2)

3. data1 <- read.table("data1.txt", sep="\t", header=T, check.names=F)

4. treatments <- read.table(textConnection(

'id description

1 "ICIs+Chemo"

2 "Chemo"

3 "ICIs"

4 "ICIs+Chemo+Antiangio"

'), header=TRUE)

5.network <- mtc.network(data.re=data1,description="PFS network", treatments=treatments)

summary(network)

plot(network, use.description = TRUE, vertex.label.cex=1.5, vertex.size=20, vertex.shape="circle", vertex.label.color="darkblue", vertex.label.dist=3, vertex.label.degree=-pi/3, vertex.color="blue", dynamic.edge.width=TRUE,edge.color="gray", vertex.label.font=2)

tiff("network.tiff", height=5000,width=5000, res= 600)

plot(network, use.description = TRUE, vertex.label.cex=1.5, vertex.size=20, vertex.shape="circle", vertex.label.color="darkblue", vertex.label.dist=3, vertex.label.degree=-pi/3, vertex.color="blue", dynamic.edge.width=TRUE,edge.color="gray", vertex.label.font=2)

dev.off()

6.model.ran <- mtc.model(network, likelihood="binom", link="logit", type = "consistency", linearModel='random',dic=TRUE)

cat(model.ran$code)

result.ran <- mtc.run(model.ran, n.adapt=10000, n.iter=500000, thin=10)

gelman.diag(result.ran)

7. gelman.plot(result.ran)

tiff("dcslzd.tiff", height=5000,width=5000, res= 600)

gelman.plot(result.ran, digits = 2)

dev.off()

8. summary(result.ran)

9.plot(result.ran)

tiff("dcgjmdt.tiff", height=5000,width=4000, res= 600)

plot(result.ran, digits = 2)

dev.off()

10. forest(relative.effect(result.ran, t1="2"), use.description = TRUE)

tiff("senlintu.tiff", height=5000,width=4000, res= 600)

p1<- forest(relative.effect(result.ran, t1="2"), use.description = TRUE)

p1

dev.off()

11.a <- round(exp(relative.effect.table(result.ran)),2)

write.csv(a, "ORR-network.csv")

12.modelume <-mtc.model(network, type="ume", n.chain=4, likelihood="binom", link="logit", linearModel="random")

resultsume <- mtc.run(modelume, n.adapt = 10000, n.iter = 200000, thin = 10)

summary(resultsume)

pdf(file="jiedianpilie2.pdf",width = 10,height = 7)

plot(b)

dev.off()

13.resultanohe <- mtc.anohe(network, n.adapt = 5000, n.iter = 100000, thin = 10, n.chain=10, likelihood="binom", link="logit",linearModel="random")

c<-summary(resultanohe)

plot(c)

pdf(file="yizhixingjianyanweixin.pdf",width = 10,height = 7)

plot(c)

dev.off()

14. ranks <- rank.probability(result.ran,preferredDirection=1)

print(ranks)

write.csv(ranks, "rank.csv")

15.tiff("nma.rank.tiff", height=5000,width=4000, res= 600)

plot(ranks,xlab='treatment', col=c("lightblue", "mistyrose", "lightcyan", "lavender"), ylab='Cumulative probability')

dev.off()

16. sucraranks <-sucra(ranks)

print(sucraranks)

plot(sucraranks)

17. data1rank <- read.table(file="data3.txt",header=TRUE)

data1rank

p<-ggplot(data1rank, aes(x=rank, y=prob, colour=treatments)) +geom_line(linewidth=2, lty="solid")

p1<-p+geom_point(size=5)+labs(x="Rank", y="Probability")+theme(axis.text = element_text(size=25),axis.title=element_text(size=28),panel.background=element_blank(),axis.line = element_line(color="black"), legend.title=element_text(size=35, color="black"),legend.position="bottom")+theme(legend.title = element_blank())+scale_color_manual(values = c('#ec1c24','#fdbd10','#0066b2','#9933CC','#FF00CC'))+theme(legend.text=element_text(family = "serif", size=20))

p1

tiff("nma.rank.tiff", height=5000,width=7000, res= 600)

p1

dev.off()

R language code for any grade AEs:

1.setwd("C:\\Users\\Administrator\\Desktop\\AEs")

2. library("gemtc")

library("rjags")

library(ggplot2)

3.data1 <- read.table("data1.txt", sep="\t", header=T, check.names=F)

4.treatments <- read.table(textConnection(

'id description

1 "ICIs+Chemo"

2 "Chemo"

3 "ICIs"

4 "ICIs+Chemo+Antiangio"

5 "Antiangio+Chemo"

'), header=TRUE)

5.network <- mtc.network(data.re=data1,description="PFS network", treatments=treatments)

summary(network)

plot(network, use.description = TRUE, vertex.label.cex=1.5, vertex.size=20, vertex.shape="circle", vertex.label.color="darkblue", vertex.label.dist=3, vertex.label.degree=-pi/3, vertex.color="blue", dynamic.edge.width=TRUE,edge.color="gray", vertex.label.font=2)

tiff("network.tiff", height=5000,width=5000, res= 600)

plot(network, use.description = TRUE, vertex.label.cex=1.5, vertex.size=20, vertex.shape="circle", vertex.label.color="darkblue", vertex.label.dist=3, vertex.label.degree=-pi/3, vertex.color="blue", dynamic.edge.width=TRUE,edge.color="gray", vertex.label.font=2)

dev.off()

6model.ran <- mtc.model(network, likelihood="binom", link="log", type = "consistency", linearModel='fixed', dic=TRUE)

cat(model.ran$code)

result.ran <- mtc.run(model.ran, n.adapt=10000, n.iter=500000, thin=10)

gelman.diag(result.ran)

7gelman.plot(result.ran)

tiff("dcslzd.tiff", height=5000,width=5000, res= 600)

gelman.plot(result.ran, digits = 2)

dev.off()

8.summary(result.ran)

9plot(result.ran)

tiff("dcgjmdt.tiff", height=5000,width=4000, res= 600)

plot(result.ran, digits = 2)

dev.off()

10 forest(relative.effect(result.ran, t1="2"), use.description = TRUE)

tiff("senlintu.tiff", height=5000,width=4000, res= 600)

p1<- forest(relative.effect(result.ran, t1="2"), use.description = TRUE)

p1

dev.off()

11.tb<- relative.effect.table(result.ran)

print(tb)

tb1<-round(exp(tb),2)

tb1

write.csv(tb1, "AE-network.csv")

12.modelume <-mtc.model(network, type="ume", n.chain=4, likelihood="binom", link="log", linearModel="fixed")

resultsume <- mtc.run(modelume, n.adapt = 10000, n.iter = 200000, thin = 10)

summary(resultsume)

pdf(file="jiedianpilie2.pdf",width = 10,height = 7)

plot(b)

dev.off()

13.resultanohe <- mtc.anohe(network, n.adapt = 5000, n.iter = 100000, thin = 10, n.chain=10, likelihood="binom", link="log", linearModel="fixed ")

c<-summary(resultanohe)

plot(c)

pdf(file="yizhixingjianyanweixin.pdf", width = 10,height = 7)

plot(c)

dev.off()

14. ranks <- rank. probability(result.ran, preferredDirection=-1)

print(ranks)

write.csv(ranks, "rank.csv")

15. tiff("nma.rank.tiff", height=5000,width=4000, res= 600)

plot(ranks,xlab='treatment', col=c("lightblue", "mistyrose", "lightcyan", "lavender"), ylab='Cumulative probability')

dev.off()

16. sucraranks <-sucra(ranks)

print(sucraranks)

plot(sucraranks)

17. data1rank <- read.table(file="data3.txt",header=TRUE)

data1rank

p<-ggplot(data1rank, aes(x=rank, y=prob, colour=treatments)) +geom_line(linewidth=2, lty="solid")

p1<-p+geom_point(size=5)+labs(x="Rank", y="Probability")+theme(axis.text = element_text(size=25),axis.title=element_text(size=28),panel.background=element_blank(),axis.line = element_line(color="black"), legend.title=element_text(size=35, color="black"),legend.position="bottom")+theme(legend.title = element_blank())+scale_color_manual(values = c('#ec1c24','#fdbd10','#0066b2','#9933CC','#FF00CC'))+theme(legend.text=element_text(family = "serif", size=20))

p1

tiff("nma.rank.tiff", height=5000,width=7000, res= 600)

p1

dev.off()
